# GLUT3 promotes macrophage signaling and function via RAS-mediated endocytosis in atopic dermatitis and wound healing

**DOI:** 10.1172/JCI170706

**Published:** 2023-11-01

**Authors:** Dong-Min Yu, Jiawei Zhao, Eunice E. Lee, Dohun Kim, Ruchika Mahapatra, Elysha K. Rose, Zhiwei Zhou, Calvin Hosler, Abdullah El Kurdi, Jun-Yong Choe, E. Dale Abel, Gerta Hoxhaj, Kenneth D. Westover, Raymond J. Cho, Jeffrey B. Cheng, Richard C. Wang

**Affiliations:** 1 Department of Dermatology, UT Southwestern Medical Center, Dallas, Texas, USA.; 2 Division of Hematology/Oncology, Boston Children’s Hospital and Department of Pediatric Oncology, Dana-Farber Cancer Institute, Harvard Medical School, Boston, Massachusetts, USA.; 3 Broad Institute of MIT and Harvard, Cambridge, Massachusetts, USA.; 4 Children’s Medical Center Research Institute and; 5 Departments of Biochemistry and Radiation Oncology, UT Southwestern Medical Center, Dallas, Texas, USA.; 6 Department of Biochemistry and Molecular Genetics, American University of Beirut, Beirut, Lebanon.; 7 Department of Chemistry and Chemical Biology, University of New Mexico, Albuquerque, New Mexico, USA.; 8 Department of Medicine, David Geffen School of Medicine, UCLA, Los Angeles, California, USA.; 9 Department of Dermatology, UCSF, San Francisco, California, USA.; 10 Harold C. Simmons Cancer Center, UT Southwestern Medical Center, Dallas, Texas, USA.

**Keywords:** Dermatology, Immunology, Glucose metabolism, Macrophages, Skin

## Abstract

The facilitative GLUT1 and GLUT3 hexose transporters are expressed abundantly in macrophages, but whether they have distinct functions remains unclear. We confirmed that GLUT1 expression increased after M1 polarization stimuli and found that GLUT3 expression increased after M2 stimulation in macrophages. Conditional deletion of *Glut3* (LysM-Cre *Glut3^fl/fl^*) impaired M2 polarization of bone marrow–derived macrophages. Alternatively activated macrophages from the skin of patients with atopic dermatitis showed increased GLUT3 expression, and a calcipotriol-induced model of atopic dermatitis was rescued in LysM-Cre *Glut3^fl/fl^* mice. M2-like macrophages expressed GLUT3 in human wound tissues as assessed by transcriptomics and costaining, and GLUT3 expression was significantly decreased in nonhealing, compared with healing, diabetic foot ulcers. In an excisional wound healing model, LysM-Cre *Glut3^fl/fl^* mice showed significantly impaired M2 macrophage polarization and delayed wound healing. GLUT3 promoted IL-4/STAT6 signaling, independently of its glucose transport activity. Unlike plasma membrane–localized GLUT1, GLUT3 was localized primarily to endosomes and was required for the efficient endocytosis of IL-4Rα subunits. GLUT3 interacted directly with GTP-bound RAS in vitro and in vivo through its intracytoplasmic loop domain, and this interaction was required for efficient STAT6 activation and M2 polarization. PAK activation and macropinocytosis were also impaired without GLUT3, suggesting broader roles for GLUT3 in the regulation of endocytosis. Thus, GLUT3 is required for efficient alternative macrophage polarization and function, through a glucose transport–independent, RAS-mediated role in the regulation of endocytosis and IL-4/STAT6 activation.

## Graphical abstract

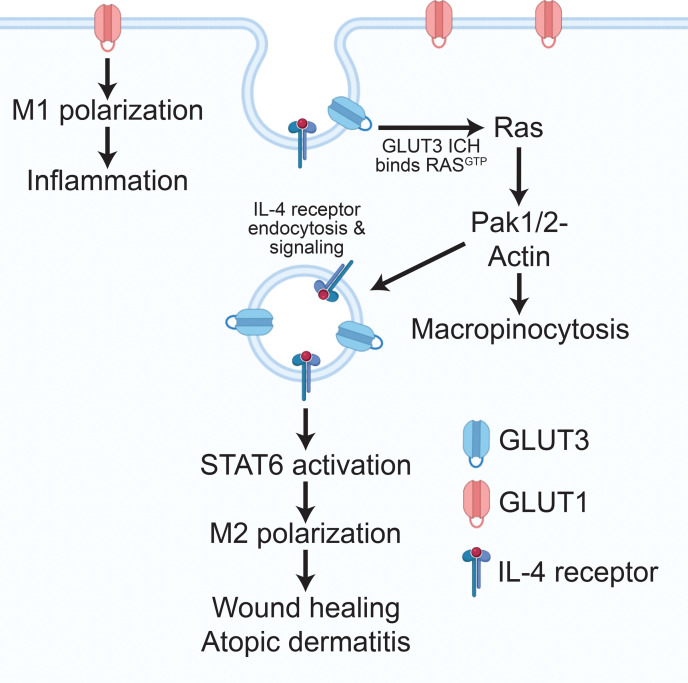

## Introduction

Macrophages are immune cells that play critical roles in both inflammation and tissue homeostasis. While macrophages appear to exhibit dynamic and complex functional phenotypes in vivo, the dichotomous model of macrophage activation remains a critical paradigm to understand macrophage functions (1). Classically activated (M1) macrophages typically promote antimicrobial and tumoricidal activity, while alternatively activated (M2) macrophages promote phagocytosis and tissue homeostasis (2, 3). M1 polarization can be induced by interferon γ (IFN-γ) and Toll-like receptor (TLR) agonists such as lipopolysaccharides (LPS), while M2 polarization is induced by IL-4 or IL-13 (4, 5). During the M1 polarization process, nuclear factor κ-light-chain enhancer of activated B cell (NF-κB) and signal transducer and activator of transcription 1 (STAT1) are activated, while M2 polarization is mainly regulated by the activation of STAT6, which then results in the expression and function of M1- and M2-specific markers, respectively. For the STAT6 signaling pathway, the binding of ligands, including IL-4, to receptors leads to the activation of Janus kinases (JAKs). Activated JAK phosphorylates receptor tyrosine residues, and phospho-tyrosine sites of these receptors serve as docking sites for STAT6 (6).

Glucose transporters are responsible for the first step of glucose utilization in cells, and 13 facilitative glucose transporters (GLUTs) are expressed in humans (7). Among them, at least GLUT1 and GLUT3 were found to be expressed abundantly in human lymphocytes and macrophages (8). Both GLUT1 and GLUT3 are class I glucose transporters, and despite high similarity in amino acid sequence and structure, the 2 transporters show differences in their pattern of tissue expression and expression levels in different cell types (9, 10). GLUT1, the most widely expressed facilitative GLUT, is highly expressed in erythrocytes, blood-brain barrier endothelial cells, and keratinocytes (11, 12). GLUT3 shows a more tissue-specific expression pattern than GLUT1 and is highly expressed in neurons and hematopoietic lineage cells (13, 14). Functional studies examining the isoform-specific functions of GLUTs in macrophages, and leukocytes in general, remain limited.

Endocytosis has traditionally been known as a mechanism to prevent excessive ligand-induced activation of downstream effectors by removing activated receptors on the cell surface (15). However, endosomes can also act as a signaling platform for numerous receptor tyrosine kinases (RTKs), including epidermal growth factor receptor (EGFR), by ensuring sufficient duration and intensity of signaling (16, 17). In particular, for IL-4 receptor (IL-4R) signaling, endosomes have been found to be essential for efficient ligand-induced receptor dimerization and signal transduction. IL-4R subunit endocytosis is distinct from the endocytosis of many RTKs and transforming growth factor β (TGF-β), and has been found to be mediated by Rac1, p21-activated kinase (PAK), and actin (18).

In this study, we evaluated the subcellular localization and function of the most highly expressed GLUTs in macrophages, GLUT1 and GLUT3. We confirmed that GLUT1 expression was increased in M1 macrophages and discovered that GLUT3 expression was increased in M2 macrophages. Notably, LysM-Cre *Glut3^fl/fl^* bone marrow–derived macrophages (BMDMs) showed defects in M2 polarization in vitro and in vivo. IL-4/STAT6 signaling, the main signaling for M2 polarization, was impaired by GLUT3 deficiency. Unlike GLUT1, which localized to the plasma membrane, GLUT3, along with components of the IL-4 signaling pathway, localized to endosomes. Finally, we found that GLUT3 interacts directly with RAS and regulates PAK activation and IL-4R endocytosis. Thus, our studies reveal that endosomal GLUT3 is essential for M2 polarization of macrophages by regulating IL-4/STAT6 signaling.

## Results

### GLUT3 is induced by M2 stimuli and required for M2 polarization of macrophages.

**Figure 1:**
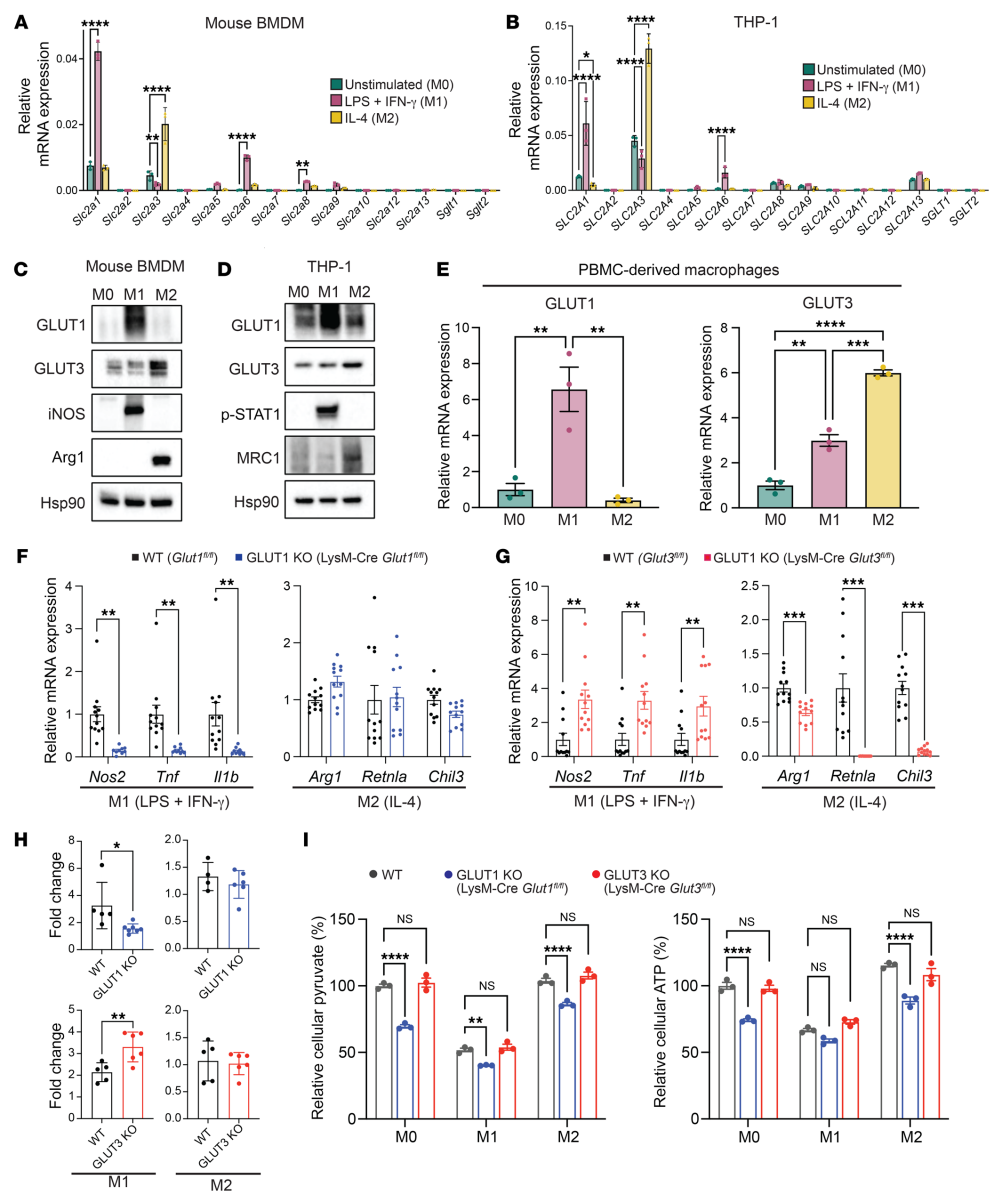
Expression of GLUT3 is increased by M2 stimulus, and GLUT3 deficiency impairs M2 polarization of macrophages. (**A** and **B**) mRNA expression levels of *GLUT* and *SGLT* transporter isoforms in BMDMs (**A**) and THP-1 cells (**B**) cells in unstimulated macrophages (green) and after treatment with classic M1 (purple) or alternative M2 (yellow) polarization stimuli for 24 hours. Expression normalized to that of β-actin (*ACTB*) (*n* = 3 biological replicates). (**C** and **D**) Western blot assessing expression of GLUT1 and GLUT3 with the indicated polarization stimuli in BMDMs (**C**) and THP-1 (**D**). iNOS and p-STAT1, M1 polarization markers; Arg1 and MRC1, M2 polarization markers; Hsp90, loading control. (**E**) GLUT1 (*Slc2a1*) and GLUT3 (*Slc2a3*) expression in primary human CD14^+^ peripheral blood monocyte–derived macrophages after treatment with indicated polarization stimuli. (**F**) mRNA expression levels of M1 (*Nos2*, *Tnfa*, and *Il1b*) and M2 (*Arg1*, *Retnla*, and *Chil3l3*) markers in WT (*n* = 12) and LysM-Cre *Glut1^fl/fl^* (GLUT1 KO) (*n* = 12) BMDMs after the indicated polarization stimuli (*n* = 4 biological replicates). (**G**) mRNA expression levels of M1 and M2 markers in WT (*n* = 12) and LysM-Cre *Glut3^fl/fl^* (GLUT3 KO) (*n* = 12) BMDMs after the indicated polarization stimuli (*n* = 4 biological replicates). (**H**) 2-Deoxy-D-glucose uptake in WT, LysM-Cre *Glut1^fl/fl^*, and LysM-Cre *Glut3^fl/fl^* BMDMs after the indicated polarization stimuli. Fold change represents uptake relative to uptake in unstimulated BMDMs from the same mouse. Data shown as mean ± SEM (*n* = 2 biological replicates). (**I**) Pyruvate and ATP levels in WT, LysM-Cre *Glut1^fl/fl^*, and LysM-Cre *Glut3^fl/fl^* BMDMs after the indicated polarization stimuli. *P* values were calculated by 2-way ANOVA with Dunnett’s test (**A** and **B**), 1-way ANOVA with Dunnett’s test (**E** and **I**), or 2-tailed *t* test (**H**). *P ≤ 0.05; **P ≤ 0.01; ***P ≤ 0.001; ****P ≤ 0.0001.

To investigate the regulation of glucose transporter (GLUT/SGLT) isoforms in macrophages, we first determined their expression levels after polarization stimuli. Mouse BMDMs were treated with LPS and IFN-γ to induce M1 polarization, and IL-4 to induce M2 polarization, and expression of facilitative (GLUT) and sodium-dependent glucose transporter (SGLT) isoforms was assessed by quantitative real-time RT-PCR (qRT-PCR). Consistent with previous studies, *Glut1* mRNA expression was elevated in M1 macrophages (19). Notably, *Glut3* mRNA expression was significantly elevated in M2 macrophages (Figure 1A). This finding was reproduced in additional macrophage cell lines, including human THP-1 cells and murine Raw 264.7 cells where GLUT1 and GLUT3 mRNA expression were similarly increased by M1 and M2 stimuli, respectively (Figure 1B and Supplemental Figure 1A; supplemental material available online with this article; https://doi.org/10.1172/JCI170706DS1). Consistent with their mRNA expression level, GLUT1 protein levels were significantly elevated after M1 polarization, while GLUT3 was significantly increased by M2 polarization stimuli in both BMDMs and THP-1 cells (Figure 1, C and D, and Supplemental Figure 1, B–D). Next, we tested whether primary human peripheral blood–derived CD14^+^ monocytes (PBMCs) cells showed similar changes. PBMC-derived macrophages significantly upregulated *GLUT1* after M1, but not M2, polarization stimuli. In contrast to BMDMs and THP-1 cells, both M1 and M2 stimuli induced *GLUT3* expression in PBMC-derived macrophages; however, its upregulation was significantly increased when compared with both uninduced and M1-polarized macrophages (Figure 1E). Because GLUT1 and GLUT3 were the 2 glucose transporters most strikingly impacted by M1 and M2 polarization stimuli, we next determined whether their expression might also affect macrophage function using genetic analyses. To assess the functional effect of GLUT1 and GLUT3 in BMDMs, mice expressing LysM-Cre recombinase were crossed with *Glut1^fl/fl^* (*Slc2a1^fl/fl^*; herein LysM-Cre *Glut1^fl/fl^*) or *Glut3^fl/fl^* (*Slc2a3^fl/fl^*; herein LysM-Cre *Glut3^fl/fl^*) mice. Consistent with previous reports, LysM-Cre *Glut1^fl/fl^* BMDMs showed significantly decreased expression of several well-established M1 markers, including nitric oxide synthase 2 (*Nos2*), tumor necrosis factor α (*Tnfa*), and IL-1β (*Il1b*) after M1 polarization stimuli. However, no significant differences in M2 markers — *Arg1* (arginase), *Retnla* (resistin-like α), and *Chil3* (chitinase-like 3) — after M2 polarization were noted between WT and LysM-Cre *Glut1^fl/fl^* BMDMs (Figure 1F). Surprisingly, LysM-Cre *Glut3^fl/fl^* BMDMs showed significantly increased expression of M1 markers (*Nos2*, *Tnfa*, and *Il1b*) compared with WT BMDMs after M1 polarization. However, the expression of other M1 activation markers (*Cd40* and *Cd86*) was not significantly changed (Figure 1G and Supplemental Figure 1D). Notably, the expression of M2 markers (*Arg1*, *Retnla*, *Chil3*, and *CD206*) was significantly reduced after M2 polarization (Figure 1G and Supplemental Figure 1E). We next investigated the effect of GLUT1 or GLUT3 deficiency on glucose uptake using 2-deoxyglucose (2-DG) uptake assays in macrophages. After M1 polarization stimuli, LysM-Cre *Glut1^fl/fl^* BMDMs showed significantly decreased glucose uptake compared with WT BMDMs. After M2 polarization, glucose uptake in LysM-Cre *Glut1^fl/fl^* macrophages decreased slightly, but these differences were not significant (Figure 1H). After M1 polarization, LysM-Cre *Glut3^fl/fl^* BMDMs showed significantly increased glucose uptake compared with WT BMDMs; this finding was consistent with the increased expression of M1 polarization markers in LysM-Cre *Glut3^fl/fl^* BMDMs. After M2 polarization, WT and LysM-Cre *Glut3^fl/fl^* BMDMs showed no differences in glucose uptake (Figure 1H). These data suggest that GLUT1, but not GLUT3, contributes primarily to glucose uptake in macrophages. To extend these findings, levels of pyruvate and ATP in WT, LysM-Cre *Glut1^fl/fl^*, and LysM-Cre *Glut3^fl/fl^* BMDMs were assessed. Notably, LysM-Cre *Glut1^fl/fl^* BMDMs showed decreased levels of pyruvate and ATP, in the nonactivated state (M0) or after M1 or M2 polarization. In contrast, LysM-Cre *Glut3^fl/fl^* BMDMs did not show differences in either ATP or pyruvate when compared to WT cells (Figure 1I). Next, we assessed glycolytic flux through ^13^C_6_-glucose and extracellular lactate measurements to assess the impact of GLUT1 or GLUT3 on glucose metabolism in macrophages. Consistent with previous reports, LysM-Cre *Glut1^fl/fl^* BMDMs showed decreased labeling of glycolytic flux (^13^C-labeled pyruvate and lactate) after M1 polarization. In contrast, LysM-Cre *Glut3^fl/fl^* BMDMs did not show significantly decreased ^13^C-labeling of metabolites under any polarization condition (Supplemental Figure 2, A–C). Finally, we further analyzed the effect of GLUT1 or GLUT3 on lactate secretion, which provides an additional measurement of glycolysis. As expected, LysM-Cre *Glut1^fl/fl^* BMDMs showed significantly decreased lactate secretion after M1 polarization, while LysM-Cre *Glut3^fl/fl^* had no effect (Supplemental Figure 2D). The knockout of GLUT1 and GLUT3 in macrophages also did not result in the compensatory upregulation of any other well-characterized glucose transporter (Supplemental Figure 3, A and B). These data support the conclusion GLUT1 is required for M1 polarization and glucose uptake, while GLUT3 promotes M2 polarization without an obvious impact on glucose transport or metabolism.

### A role for myeloid GLUT3 in allergic inflammation and atopic dermatitis.

**Figure 2:**
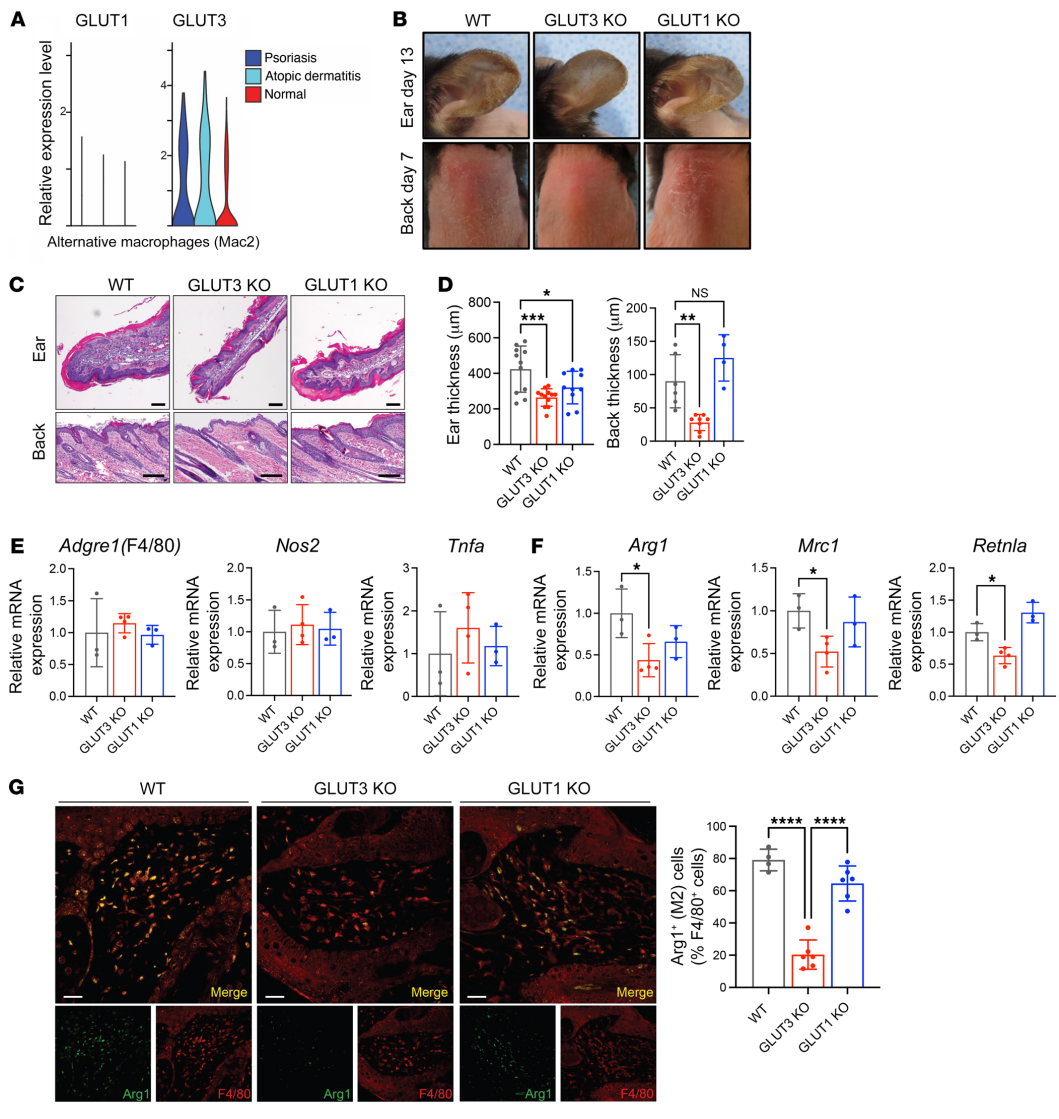
Myeloid LysM-Cre *Glut3^fl/fl^* (GLUT3 KO) rescues a mouse model of calcipotriol-induced inflammation. (**A**) Violin plots showing relative expression of GLUT1 and GLUT3 in alternative macrophages (Mac2) from single-cell RNA-seq profiles of CD45^+^ cells from patients with psoriasis vulgaris (*n* = 8), atopic dermatitis (*n* = 7), or healthy controls (*n* = 7). (**B**) Representative photos after calcipotriol administration in WT, LysM-Cre *Glut3^fl/fl^* (GLUT3 KO), and LysM-Cre *Glut1^fl/fl^* (GLUT1 KO) mice on day 8. (**C**) Hematoxylin and eosin–stained sections of mouse skin treated with calcipotriol analyzed on day 13 in WT, LysM-Cre *Glut3^fl/fl^*, and LysM-Cre *Glut1^fl/fl^* mice. Scale bars: 100 μm. (**D**) Thickness of calcipotriol-treated ear and back in WT (*n* = 11 for ear and *n* = 6 for back), LysM-Cre *Glut3^fl/fl^* (*n* = 12 for ear and *n* = 8 for back), and LysM-Cre *Glut1^fl/fl^* (*n* = 10 for ear and *n* = 4 for back) mice. (**E** and **F**) mRNA expression levels in calcipotriol-treated ear in WT (*n* = 3), LysM-Cre *Glut3^fl/fl^* (*n* = 4), and LysM-Cre *Glut1^fl/fl^* (*n* = 3) mice. Pan-macrophage marker (*Adgre1* [F4/80]), M1 markers (*Nos2* and *Tnfa*) (**E**), and M2 markers (*Arg1*, *Mrc1*, and *Retnla*) (**F**) were observed. (**G**) Representative immunofluorescent staining of Arg1 (green) and F4/80 (red) in the back skin of calcipotriol treated mice (day 8). Scale bars: 50 μm. (Right) Quantification of M2 macrophages at wound sites (day 8). The number of F4/80^+^Arg1^+^ (M2) cells present relative to the total number of F4/80^+^ cells. Data shown as mean ± SEM. *P* values were calculated by 1-way ANOVA with Dunnett’s test. *P ≤ 0.05; **P ≤ 0.01; ***P ≤ 0.001; ****P ≤ 0.0001.

Given the striking impact of GLUT3 deficiency on M2 polarization in vitro, we next investigated its effect on macrophage function in vivo. While M2 macrophages are primarily thought to function in tissue homeostasis, they have also been shown to play important roles in promoting allergic (type 2) inflammation (20, 21). The single-cell transcriptomes of CD45^+^ immune cells from patients with atopic dermatitis or psoriasis were reanalyzed for the expression of GLUT1 and GLUT3 (22). Of the multiple macrophage populations identified in vivo, 1 population, Mac2, expressed markers consistent with alternative macrophage differentiation, including expression of *NR4A1*, *NR4A2*, and *KLF4* (23). The expression of GLUT3, but not GLUT1, was 2.5-fold higher in alternative macrophages (Mac2) in patients with atopic dermatitis compared with healthy controls (Figure 2A). Biopsies from patients with atopic dermatitis were assessed for the expression of GLUT3 by immunofluorescence (IF). Specifically, costaining of atopic dermatitis biopsies for the human monocyte marker, CD68, and GLUT3 revealed numerous CD68^+^GLUT3^+^ double-positive cells in the papillary dermis of the affected tissues (Supplemental Figure 4A, arrows). To determine whether GLUT3 had a functional role in M2 macrophages, we assessed the impact of deletion of macrophage GLUT3 in a mouse model of an atopic dermatitis–like rash. Dermatitis was induced in WT, LysM-Cre *Glut3^fl/fl^*, and LysM-Cre *Glut1^fl/fl^* mice through the topical administration of calcipotriol (MC903), and the development of the inflammation was assessed (24, 25) (Supplemental Figure 4B). The back and treated ears of WT and LysM-Cre *Glut1^fl/fl^* mice showed notable edema, erythema, and scaling, consistent with previous descriptions of the dermatitis induced by calcipotriol. LysM-Cre *Glut3^fl/fl^* mice showed notably less inflammation, with decreased edema, erythema, and scale compared with WT and LysM-Cre *Glut1^fl/fl^* mice (Figure 2, B and C). Consistent with the macroscopic appearance, histological analyses revealed decreased epidermal hyperplasia and hyperkeratosis in LysM-Cre *Glut3^fl/fl^* mice compared with LysM-Cre *Glut1^fl/fl^* mice or WT mice (Figure 2D). Calcipotriol-treated tissues were harvested (day 13) and qRT-PCR was used to detect markers of macrophage polarization. The expression of *Adgre1* (F4/80), a pan-macrophage marker, was similar in WT, LysM-Cre *Glut3^fl/fl^*, and LysM-Cre *Glut1^fl/fl^* mice (Figure 2E). Moreover, calcipotriol-treated tissues showed no significant changes in M1 markers, including *Nos2* and *Tnfa*, between WT, LysM-Cre *Glut3^fl/fl^*, and LysM-Cre *Glut1^fl/fl^* mice (Figure 2E). However, several M2 markers, including *Arg1*, *Mrc1*, and *Retnla*, were significantly decreased in LysM-Cre *Glut3^fl/fl^* mice compared with WT and LysM-Cre *Glut1^fl/fl^* mice (Figure 2F). In addition, the levels of several Th2 cytokines, including *Il4*, *Il13*, and *Il31*, which have been shown to increase in response to calcipotriol treatment (24, 26), were significantly reduced in LysM-Cre *Glut3^fl/fl^* compared with WT mice (Supplemental Figure 4C). One limitation of these qRT-PCR data is that they assess expression from a heterogeneous cell population; thus, we performed colocalization experiments to confirm the impact of GLUT3 on M2-like markers in calcipotriol-induced dermatitis. Indeed, the knockout of GLUT3, but not GLUT1, resulted in a significant decrease in the percentage of F4/80^+^Arg1^+^ double-positive M2-like cells after calcipotriol treatment (Figure 2G).

### A role for macrophage GLUT3 in wound healing.

**Figure 3:**
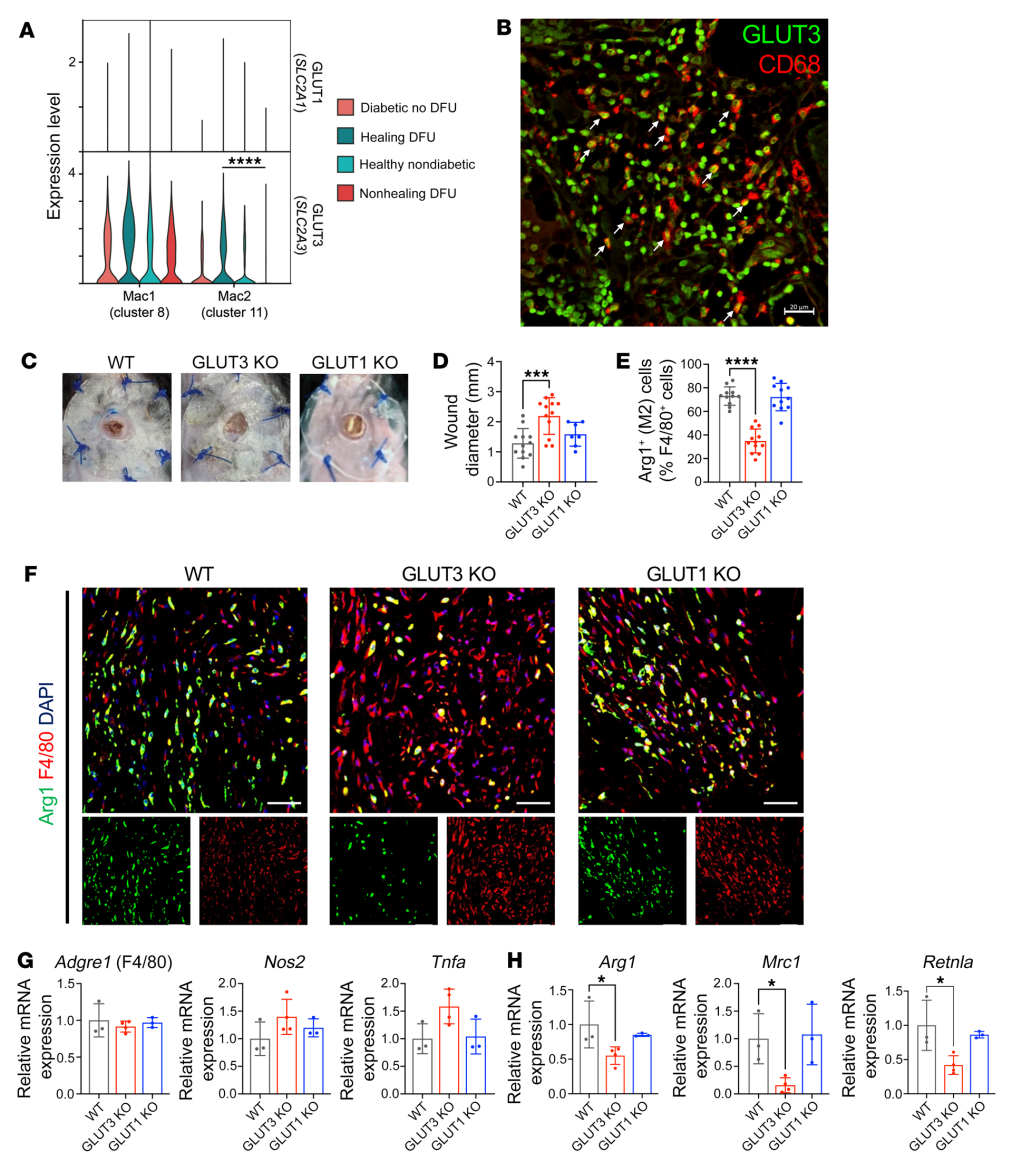
Delayed wound healing in LysM-Cre *Glut3^fl/fl^* (GLUT3 KO) mice. (**A**) Violin plots showing relative expression of *GLUT1* and *GLUT3* in alternative macrophages (Mac2) from single-cell RNA-seq profiles of indicated tissues from diabetic patients with no foot ulcer (*n* = 6), healing diabetic foot ulcer (*n* = 7), healthy nondiabetic skin (*n* = 10), or nonhealing diabetic foot ulcer (*n* = 4). Transcriptomic data are from Theocharidis et al. (28). DFU, diabetic foot ulcer. (**B**) Representative immunofluorescent staining of a patient biopsy specimen of a wound bed for CD68 (red) and GLUT3 (green). Arrows indicate cells expressing both CD68 and GLUT3 in the wound bed (see Supplemental Figure 5A). Scale bar: 20 μm. (**C**) Representative photos of wound site in WT, LysM-Cre *Glut3^fl/fl^* (GLUT3 KO), and LysM-Cre *Glut1^fl/fl^* (GLUT1 KO) mice 6 days after injury. (**D**) Measurements of wound diameter on day 6 in WT (*n* = 12), LysM-Cre *Glut3^fl/fl^* (*n* = 12), and LysM-Cre *Glut1^fl/fl^* (*n* = 7) mice (see Supplemental Figure 5C). (**E**) Quantification of M2 macrophages at wound sites (day 6). The number of F4/80^+^Arg1^+^ (M2) cells present relative to the total number of F4/80^+^ cells (see Supplemental Figure 5D). (**F**) Representative immunofluorescent staining for Arg1 (green), F4/80 (red), and with DAPI (blue) in the wound site (day 6). Scale bars: 50 μm. (**G** and **H**) mRNA expression levels of M0/M1 markers (*Adgre1* [F4/80], *Nos2*, and *Tnfa*) (**E**) or M2 markers (*Arg1*, *Mrc1*, and *Retnla*) (**F**) in WT (*n* = 3), LysM-Cre *Glut3^fl/fl^* (*n* = 3), and LysM-Cre *Glut1^fl/fl^* (*n* = 3) mice. Data shown as mean ± SEM. *P* values were calculated by 1-way ANOVA with Dunnett’s test. *P ≤ 0.05; ***P ≤ 0.001; ****P ≤ 0.0001.

M2 macrophages also play critical roles in wound healing, particularly in the later phases of neovascularization and tissue remodeling (27). Thus, we analyzed the single-cell transcriptomes of tissues from the wounds of patients with diabetic foot ulcers for the expression of *GLUT1* and *GLUT3* (28). While *IL1B*^+^ M1-like and *CD163*^+^ M2-like macrophage populations were identified, extensive overlap between the expression of in vitro polarization markers was noted in the 2 populations, consistent with the observation that macrophage polarization occurs on a spectrum in vivo (3). In wounded tissues, *GLUT3* expression was increased in M2-like macrophages from healing diabetic foot ulcers compared with normal skin, and *GLUT3* expression was significantly decreased in this population in nonhealing diabetic foot ulcers, suggesting a functional role for GLUT3 in wound healing (Figure 3A). *GLUT3* expression was also noted in the M1-like macrophages, and its expression was also significantly decreased in this population in nonhealing wounds. Colocalization experiments in healing, wounded tissues were conducted to confirm the transcriptomic analyses. Costaining of healing wounds for CD68 and GLUT3 revealed numerous CD68^+^GLUT3^+^ double-positive cells in the wound bed adjacent to the wound edge (Figure 3B and Supplemental Figure 5A). To determine whether GLUT3 in M2 macrophages functioned in wound healing, we used a splinted, excisional wound healing model. The back skin of WT, LysM-Cre *Glut3^fl/fl^*, and LysM-Cre *Glut1^fl/fl^* mice was excised by punch biopsy and splinted with a silicone ring; wound diameter was measured every 2 days (Figure 3C and Supplemental Figure 5B). In LysM-Cre *Glut3^fl/fl^* mice, wound healing was significantly delayed compared with WT mice (Figure 3D and Supplemental Figure 5C). IF of the wounded tissues was used to assess the effect of LysM-Cre *Glut3^fl/fl^* on macrophage phenotype. Wounded tissues were costained for the pan-macrophage marker, F4/80, and the M2 marker, Arg1. There was no difference in the number of F4/80^+^ cells in WT, LysM-Cre *Glut1^fl/fl^*, and LysM-Cre *Glut3^fl/fl^* mice (Supplemental Figure 5D). However, the percentage of F4/80^+^Arg1^+^ double-positive (M2) macrophages was significantly reduced in LysM-Cre *Glut3^fl/fl^* mice compared with WT and LysM-Cre *Glut1^fl/fl^* (Figure 3, E and F). We also examined macrophage markers by qRT-PCR in tissue from the wound edge to corroborate the IF experiments. Total RNA was obtained from the wound edge on day 6. Consistent with the in vitro findings, there were no differences in the mRNA expression level of total (*Adgre1* [F4/80]) and M1 (*Nos2*, *Tnfa*) macrophage markers (Figure 3G), but the expression of the M2 markers (*Arg1*, *Mrc1*, *Retnlna*) was significantly reduced in LysM-Cre *Glut3^fl/fl^* mice (Figure 3H). Specific markers previously implicated in tissue remodeling (*Tgfb*, *Acta2*, *Col3a1*) were also significantly reduced in LysM-Cre *Glut3^fl/fl^* mice (29–31). Consistent with a reported role for both M1 and M2 macrophages in distinct aspects of angiogenesis (32), reductions in several markers relevant to angiogenesis were decreased in both LysM-Cre *Glut1^fl/fl^* and LysM-Cre *Glut3^fl/fl^* mice, but the differences were significant in LysM-Cre *Glut3^fl/fl^* (*Vegfa*, *Tek*, *Cxcr3*) (Supplemental Figure 5, E–F). Our results demonstrate that GLUT3 plays a critical role in promoting both allergic inflammation and wound healing functions of macrophages.

### GLUT3, but not its transport activity, is required for STAT6 signaling.

**Figure 4:**
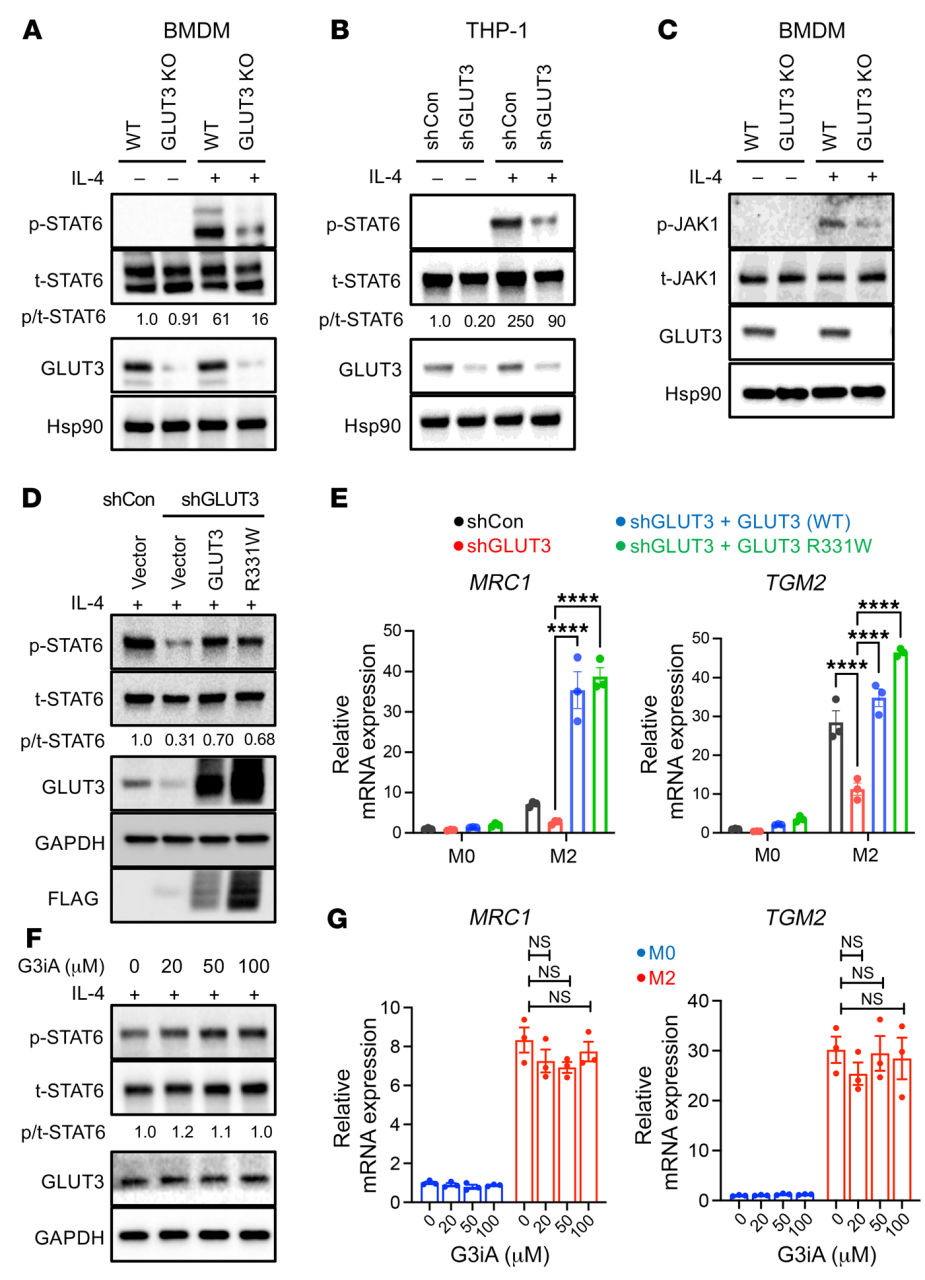
GLUT3 activates STAT6 signaling and M2 polarization independently of glucose transport activity. (**A**) Western blot (WB) for phospho-STAT6 (p-STAT6) and total STAT6 (t-STAT6) with and without IL-4 activation (30 minutes) in WT and LysM-Cre *Glut3^fl/fl^* (GLUT3 KO) BMDMs. Mean of p-STAT6/t-STAT6 levels from quantification of WB (*n* = 3 biological replicates; Supplemental Figure 6B). (**B**) Western blot for p-STAT6 and t-STAT6 after shRNA knockdown of endogenous GLUT3 in THP-1 cells (*n* = 3 biological replicates; Supplemental Figure 6C). (**C**) Levels of p-JAK1 (Y1034/1035) and t-JAK1 was determined in WT and LysM-Cre *Glut3^fl/fl^* BMDMs with and without IL-4 stimulation (*n* = 2 biological replicates). (**D**) Levels of p-STAT6 and t-STAT6 after overexpression of WT GLUT3 or GLUT3 R331W mutant and GLUT3 shRNA in THP-1 cells. Mean of p-STAT6/t-STAT6 levels from quantification of WB (*n* = 3 biological replicates; Supplemental Figure 7D). (**E**) Expression of the indicated mRNA was assessed in THP-1 cells with and without IL-4 stimulation (24 hours). (**F**) Levels of p-STAT6 and t-STAT6 were assessed in THP-1 cells after treatment with the indicated concentration of G3iA and IL-4 stimulation (30 minutes). Mean of p-STAT6/t-STAT6 levels from quantification of WB (*n* = 3 biological replicates; Supplemental Figure 7G). (**G**) Expression of the indicated mRNA was assessed in THP-1 cells with the indicated concentration of G3iA with and without IL-4 stimulation (24 hours). *P* values were calculated by 2-way ANOVA with Tukey’s test. ****P ≤ 0.0001.

To determine how GLUT3 promoted M2 polarization and function, we first assessed signaling downstream of IL-4. Notably, phosphorylation of STAT6 (p-STAT6) by IL-4 was significantly reduced in LysM-Cre *Glut3^fl/fl^* BMDMs and GLUT3 shRNA–treated THP-1 cells (Figure 4, A and B, and Supplemental Figure 6, B and C). GLUT3 knockdown also strongly reduced the expression of p-STAT6 in Raw 264.7 mouse macrophages (Supplemental Figure 6D). There was no difference in the induction of STAT1 phosphorylation (p-STAT1) by LPS and IFN-γ in LysM-Cre *Glut1^fl/fl^* BMDMs (Supplemental Figure 6A). In LysM-Cre *Glut3^fl/fl^* BMDMs, p-STAT1 was slightly increased after M1 stimuli (Supplemental Figure 6A). The effects on STAT6 signaling were confirmed in human and mouse macrophage cell lines. Consistent with JAK1’s role in phosphorylating STAT6 in response to IL-4 stimulation, levels of p-JAK1 were also decreased by GLUT3 deficiency in BMDMs (Figure 4C). Thus, GLUT3 is required for M2 polarization through its promotion of JAK1/STAT6 signaling in M2 polarization.

LysM-Cre *Glut3^fl/fl^* BMDMs did not show decreased glucose uptake, cellular pyruvate levels, or ATP levels compared to WT control. Thus, we tested whether the glucose transport function of GLUT3 was required for its role in activating STAT6. We first inhibited GLUT3-mediated transport genetically. Multiple missense mutations can block GLUT1-mediated glucose transport without affecting protein stability. Because GLUT1 and GLUT3 are highly conserved in many regions, we generated orthologous missense mutations in GLUT3 (9, 33). Lentiviral transduction of WT, shRNA-resistant, or transport-mutant alleles of GLUT3 in Rat2 fibroblasts resulted in the stable overexpression and plasma membrane localization of GLUT3 (Supplemental Figure 7, A and B). 2-DG uptake assays revealed that the R331W mutant showed the greatest reduction in glucose uptake compared with WT GLUT3, and it was selected for separation-of-function studies (Supplemental Figure 7C). shRNA–resistant alleles of both WT and transport-defective GLUT3 (R331W) were generated and stably expressed in THP-1 cells. Then, shGLUT3 was used to knock down endogenous GLUT3 in THP-1 cells, which already expressed shRNA-resistant WT or R331W mutant GLUT3. While shGLUT3 significantly decreased p-STAT6 levels, both WT and R331W GLUT3 rescued IL-4–induced p-STAT6 activation after endogenous GLUT3 knockdown (Figure 4D and Supplemental Figure 7D). We next determined whether GLUT3 transport activity would be required for the expression of M1 and M2 polarization makers. THP-1 cells expressing shRNA-resistant WT or R331W GLUT3 and shGLUT3 were treated with M1 or M2 polarization stimuli for 24 hours. Both WT and R331W GLUT3 rescued M2 marker (*MRC1* and *TGM2*) expression similarly (Figure 4E and Supplemental Figure 7E). As expected, the expression of an M1 marker (*CXCL10*) was not affected by WT or R331W GLUT3 after M1 polarization (24 hours) (Supplemental Figure 7F).

The transport-independent role of GLUT3 in STAT6 signal transduction and M2 polarization was confirmed through the chemical inhibition of GLUT3. THP-1 cells were treated with the small molecule G3iA, which potently inhibits glucose transport by GLUT3 (IC_50_ ~7 μM) but inhibits transport by closely related GLUTs (GLUT1/4/5) with lower affinity (IC_50_ > 50 μM) (34). Consistent with a transport-independent function for GLUT3 in IL-4 signal transduction, the activation of p-STAT6 by IL-4 was not impaired in THP-1 cells treated with G3iA (Figure 4F and Supplemental Figure 7G). We next determined the effect of G3iA on the expression of markers of macrophage polarization. THP-1 cells were treated with M1 or M2 polarization stimuli in the presence of increasing concentrations of G3iA for 24 hours. Inhibition of GLUT3 transport did not significantly change the expression of either M2 (*MRC1*, *TGM2*) or M1 (*CXCL10*) differentiation markers (Figure 4G and Supplemental Figure 7, H and I). Thus, GLUT3, but not its glucose transport activity, is required for optimal IL-4 signal transduction and M2 polarization.

### GLUT3 and p-STAT6 localize to endosomes.

**Figure 5:**
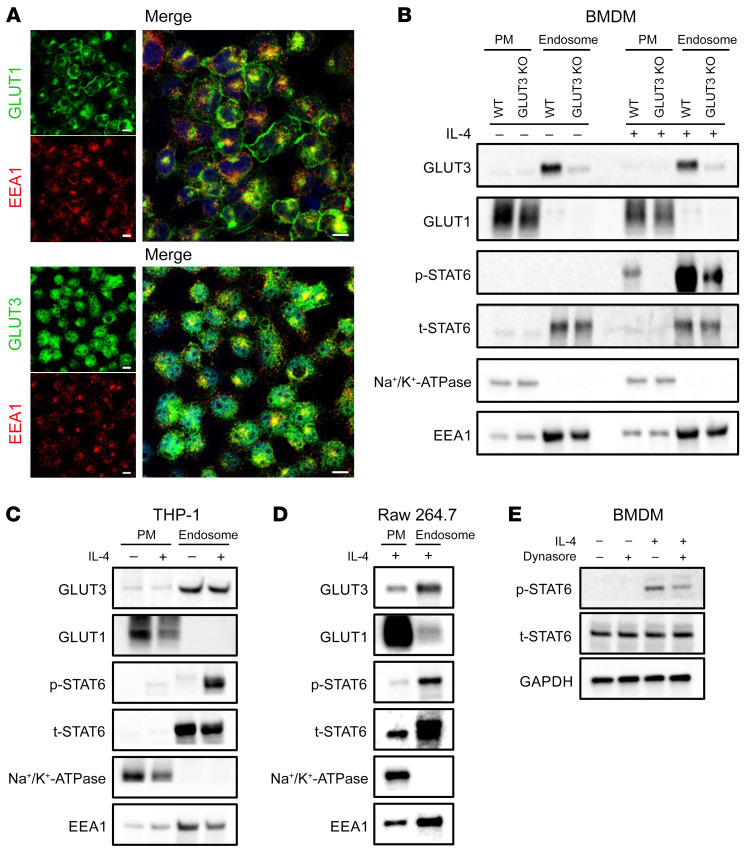
GLUT3 is localized in endosomes. (**A**) Representative immunofluorescence image of THP-1 cells labeled for GLUT1 (upper panel, green), GLUT3 (lower panel, green), EEA1 (red), and with DAPI (blue). Scale bars: 10 μm. (**B**–**D**) Western blot analysis of the expression of GLUT3, GLUT1, p-STAT6, and STAT6 in the isolated plasma membrane (PM) and endosome fraction from WT and LysM-Cre *Glut3^fl/fl^* (GLUT3 KO) BMDMs (**B**), THP-1 cells (**C**), and Raw 264.7 cells (**D**). Na^+^/K^+^-ATPase and EEA1 are fractionation controls for the plasma membrane and endosome, respectively. (**E**) Western blot analysis of the expression of p-STAT6 and t-STAT6 in BMDMs with and without IL-4 (30 minutes) and with and without Dynasore.

GLUT isoform function can be regulated through their specific localization to specific membrane compartments. Previous studies have demonstrated that GLUT3 is localized primarily intracellularly rather than at the cell surface (35, 36). For example, in primary cortical neurons, GLUT3 is mostly localized to endosomes (35). Previous studies have also demonstrated that the IL-4R/IL-4 complex undergoes endocytosis and that these endosomes play a role as signaling platforms (18). Thus, we investigated the cellular localization of GLUT3 in macrophages. IF studies of THP-1 cells and BMDMs revealed that GLUT1 localized predominantly to the plasma membrane, whereas GLUT3 minimally stained the plasma membrane and instead showed strong intracellular staining that colocalized partially with an endosomal marker, early endosome antigen 1 (EEA1) (Figure 5A and Supplemental Figure 8A). The IF was corroborated through cell fractionation experiments (Supplemental Figure 8B). The localization of GLUT1 and GLUT3 was assessed after biochemical fractionation of WT and LysM-Cre *Glut3^fl/fl^* BMDMs. While most GLUT1 was present in the plasma membrane fraction, GLUT3 was found predominantly in the endosomal fraction. These experiments also confirmed that both p-STAT6 and total STAT6 (t-STAT6) were enriched in the endosomal fraction; p-STAT6 activation was markedly impaired in LysM-Cre *Glut3^fl/fl^* BMDMs despite the presence of t-STAT6 in endosomes (Figure 5B). Fractionation experiments in THP-1 and Raw 264.7 cells also demonstrated that GLUT1 was found mostly in the plasma membrane fraction, whereas GLUT3 was predominantly present in the endosomes (Figure 5, C and D). In all macrophage cell lines, p-STAT6 was detected in endosomes only after IL-4 stimulation, while t-STAT6 and GLUT3 were detected in endosomes regardless of IL-4 stimulation (Figure 5, B and D). Consistent with a critical role for endocytosis in IL-4 signal transduction in both THP-1 cells and BMDMs, p-STAT6 induction after IL-4 stimulation (30 minutes) was significantly inhibited by Dynasore, a small molecule inhibitor of dynamin and endocytosis (Figure 5E and Supplemental Figure 8C). LysM-Cre *Glut3^fl/fl^* did not affect total endosome abundance, as assessed by quantification of EEA1 abundance (Supplemental Figure 8D). In contrast to GLUT1, GLUT3 and activated p-STAT6 both localize to endosomes, and endocytosis is necessary for efficient p-STAT6 activation.

### GLUT3 promotes IL-4R endocytosis through a direct interaction with RAS.

**Figure 6:**
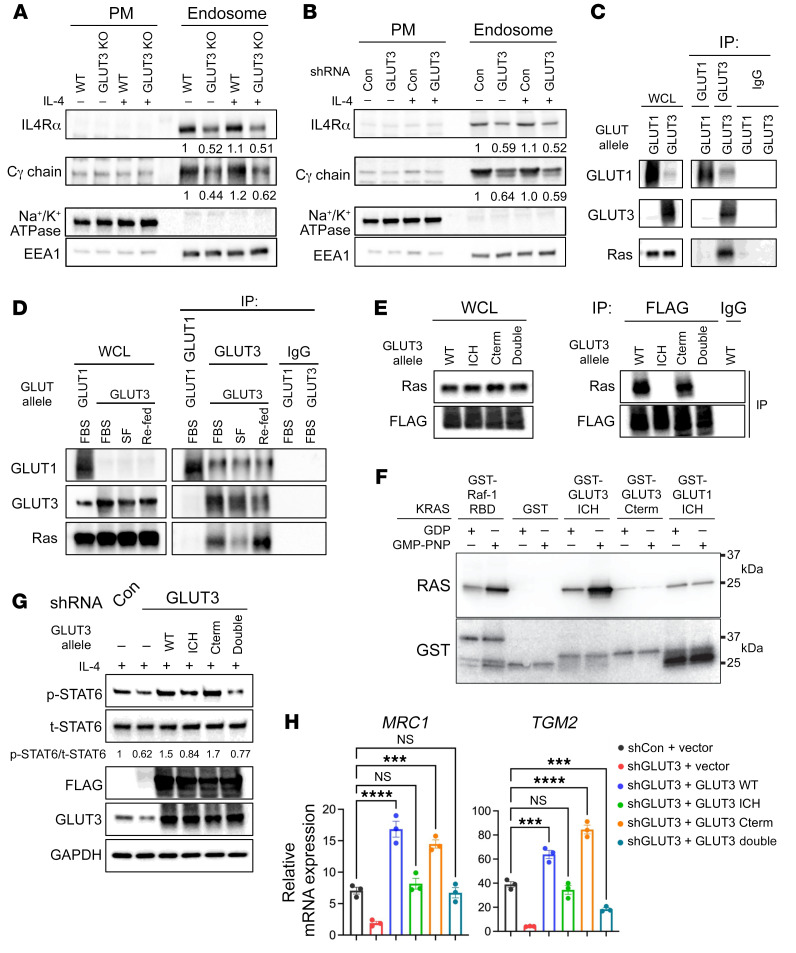
GLUT3 promotes IL-4R subunit endocytosis and M2 polarization through its interaction with RAS. (**A** and **B**) Western blot (WB) of IL-4Rα and γ_c_ chain in the plasma membrane (PM) and endosomal fractions from WT, LysM-Cre *Glut3^fl/fl^* (GLUT3 KO) BMDMs (**A**), and THP-1 cells transduced with control or GLUT3 shRNA (**B**). Na^+^/K^+^-ATPase and EEA1, fractionation controls. Mean of IL-4Rα and γ_c_ chain levels relative to EEA1 from quantification of WB (*n* = 3 biological replicates; Supplemental Figure 9, C and F). (**C**) HEK293T cells were transfected with the indicated GLUT allele, and GLUT1 (Thermo Fisher Scientific, MA1-37783) or GLUT3 (Abcam, ab15311) was immunoprecipitated. RAS was detected by Western blotting. Normal mouse/rabbit IgG, IP controls. (**D**) HEK293T cells were transfected with the indicated GLUT allele and immunoprecipitation performed after serum starvation (serum-free, SF) or refeeding as indicated. (**E**) HEK293T cells were transfected with the indicated GLUT3 allele (Supplemental Figure 9I) and GLUT3 alleles were FLAG immunoprecipitated; RAS was detected by Western blotting. IgG indicates a normal mouse IgG control. (**F**) The indicated GST fusion protein was bound to glutathione-agarose and incubated with GDP- or GTP-bound (GMP-PNP) KRAS as indicated. Bound proteins were eluted and assessed by Western blotting. The GST blot was stripped and probed for RAS to ensure even loading. (**G**) Levels of p-STAT6 after expression of indicated shRNA-resistant GLUT3 allele and shRNA of endogenous GLUT3 in THP-1 cells. (**H**) THP-1 were transduced with the indicated shRNA and shRNA-resistant GLUT3 allele and then the indicated M2 marker (*MRC1*, *TGM2*) was assessed by qRT-PCR after IL-4 stimulation (24 hours). *P* values were calculated by 1-way ANOVA with Dunnett’s test. ***P ≤ 0.001, ****P ≤ 0.0001.

Given the critical role for endocytosis in IL-4 signal transduction, we tested whether GLUT3 was required for IL-4R subunit endocytosis. Type I IL-4Rs are formed by heterodimers of IL-4Rα and the common γ (γ_c_) chain. WT and LysM-Cre *Glut3^fl/fl^* BMDMs were fractionated, and plasma membrane and endosomal preparations were prepared both with and without IL-4 treatment (30 minutes). Western blotting and qRT-PCR analyses of IL-4Rα and γ_c_ chain (*Il2rg*) confirmed that their expression was not affected by GLUT3 deficiency (Supplemental Figure 9, A and B). The endocytosis of both IL4Rα and γ_c_ was significantly reduced in LysM-Cre *Glut3^fl/fl^* BMDMs (Figure 6A and Supplemental Figure 9C). Similarly, control or shGLUT3 treatment of THP-1 cells did not affect IL-4Rα or γ_c_ mRNA or protein levels (Supplemental Figure 9, D and E), but GLUT3 knockdown impaired both basal and IL-4–induced IL-4Rα and γ_c_ endocytosis (Figure 6B and Supplemental Figure 9F). LPS signals through TLR4 to activate NF-κB and promote M1 differentiation. To determine whether GLUT3 might also affect TLR4 endocytosis, we assessed the impact of LysM-Cre *Glut3^fl/fl^* on the endosomal localization of TLR4. Indeed, TLR4 endocytosis was not decreased in LysM-Cre *Glut3^fl/fl^* BMDMs (Supplemental Figure 9G). Thus, consistent with the observation that LysM-Cre *Glut3^fl/fl^* did not impair M1 polarization, LysM-Cre *Glut3^fl/fl^* did not impair TLR4 endocytosis. The endocytosis of the γ_c_ chain is clathrin independent and regulated by small GTPases, such as RAS and RAC1 (37–40). To dissect how endosomal GLUT3 might regulate IL-4Rα and γ_c_ endocytosis, we examined how GLUT3 might regulate proteins implicated in non-clathrin-mediated endocytosis. Because proteomic studies have previously demonstrated an interaction between GLUT3 and the RAS GTPases (HRAS, NRAS, and KRAS) (41), we tested whether GLUT3 might interact with RAS in macrophages. GLUT3 coimmunoprecipitated RAS in BMDMs with and without IL-4 stimulation (Supplemental Figure 9H). In transfected HEK293T cells, GLUT3, but not GLUT1, coimmunoprecipitated RAS, confirming an interaction between RAS and GLUT3 (Figure 6C). Growth factors promote RAS activation and its binding to downstream effectors. Thus, we tested whether the interaction between GLUT3 and RAS might be regulated by serum. Indeed, the coimmunoprecipitation of GLUT3 was diminished by serum starvation and was promoted after serum refeeding (Figure 6D). GLUT1 and GLUT3 are highly homologous proteins, with most differences localizing to their intracytoplasmic loop (ICH) and carboxy terminal (Cterm) motifs. To localize the potential RAS interaction domain of GLUT3 more precisely, we generated GLUT3/GLUT1 chimeric mutants that possessed either the GLUT1 ICH, GLUT1 Cterm, or both GLUT1 domains (Supplemental Figure 9I). WT GLUT3 and the Cterm GLUT3/1 chimera interacted strongly with RAS, but not the ICH and double mutant GLUT3/1 chimeras, indicating that the GLUT3 ICH motif was necessary for RAS binding (Figure 6E). To determine whether the interaction between the GLUT3 ICH and RAS was direct, we generated GST fusions proteins of the GLUT3 ICH, GLUT3 Cterm, and GLUT1 ICH domains and expressed them in bacteria. As expected, the RAS-binding domain (RBD) of Raf-1 bound to purified KRAS in a GTP-dependent manner. Notably, the GST-GLUT3 ICH fusion also interacted with purified KRAS in a GTP-dependent manner (Figure 6F). These results suggest that the GLUT3 ICH domain binds directly to active, GTP-bound KRAS. The chimeric GLUT3/GLUT1 constructs were then tested for their ability to rescue STAT6 activation and M2 polarization after GLUT3 shRNA. shRNA–resistant WT and chimeric GLUT3 alleles were lentivirally transduced in THP-1 cells. As expected, shGLUT3 inhibited STAT6 activation in response to a 30-minute treatment with IL-4. STAT6 (p-STAT6/t-STAT6) was significantly rescued by WT and Cterm GLUT3/1 alleles, but not by the GLUT3/1 ICH and double chimeras (Figure 6G and Supplemental Figure 9J). Finally, after IL-4 stimulation, M2 polarization markers (*MRC1*, *TGM2*) were more strongly induced by WT and the Cterm GLUT3/1 chimera than by ICH and double GLUT3/1 chimeras (Figure 6H and Supplemental Figure 6K). We conclude that the GLUT3 ICH domain interacts directly with GTP-bound RAS to promote IL-4R subunit endocytosis, STAT6 signaling, and M2 polarization.

### GLUT3 is required for efficient macropinocytosis in macrophages.

**Figure 7:**
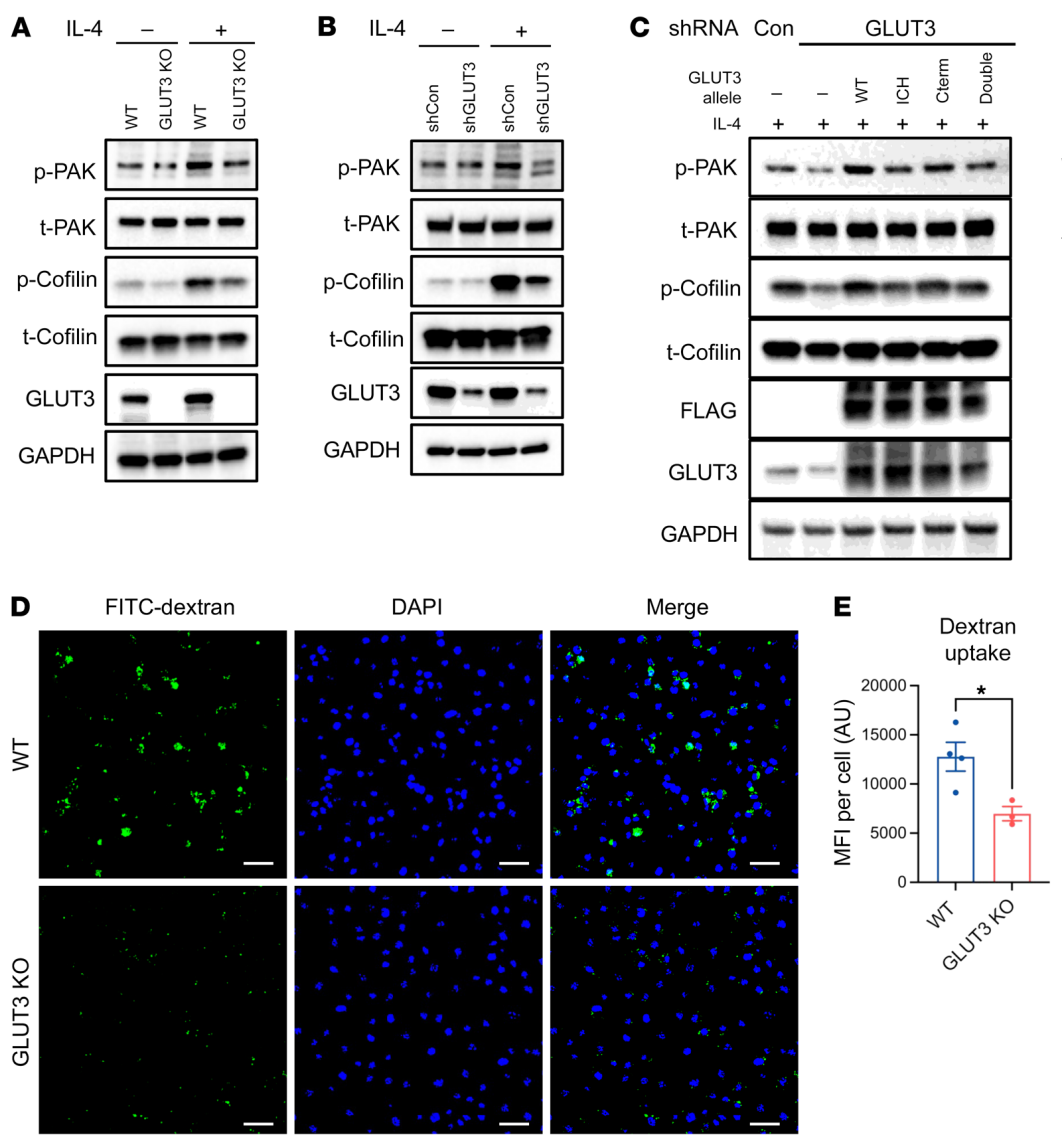
GLUT3 is required for PAK/cofilin signaling and macropinocytosis in macrophages. (**A** and **B**) Levels of phospho-PAK (p-PAK), total PAK (t-PAK), p-cofilin, and t-cofilin with and without IL-4 (30 minutes) in WT, LysM-Cre *Glut3^fl/fl^* (GLUT3 KO) BMDMs (**A**), and shRNA-transduced THP-1 cells (**B**). (**C**) Levels of p-PAK, t-PAK, p-cofilin, and t-cofilin after expression of indicated shRNA-resistant GLUT3 allele and shRNA of endogenous GLUT3 in THP-1 cells. (**D**) Representative IF image of WT or LysM-Cre *Glut3^fl/fl^* BMDMs incubated in FITC-dextran. Scale bars: 50 μm. (**E**) Quantification of mean fluorescence intensity (MFI) per field (Zen) normalized by cell number (*n* = 3 biological replicates). Data shown as mean ± SEM. *P* values were calculated by 2-tailed *t* test. *P ≤ 0.05.

One prominent downstream target of small GTPases, including RAS, are PAKs, which are also required for IL-2Rβ and γ_c_ endocytosis (37, 42, 43). We found that IL-4 stimulation activated PAK, as evidenced by increased p-PAK levels, but this activation was decreased in LysM-Cre *Glut3^fl/fl^* BMDMs and shGLUT3 THP-1 cells (Figure 7, A and B). PAK regulates endocytosis through actin remodeling by factors including cofilin (actin depolymerizing factor) (44). Cofilin phosphorylation was also activated by IL-4 stimulation and this activation was inhibited by LysM-Cre *Glut3^fl/fl^* or shRNA (Figure 7, A and B). We also explored the effects of the chimeric GLUT1/GLUT3 constructs on PAK and cofilin activation. Consistent with its effects on STAT6 activation, phosphorylation of PAK1/2 and cofilin was increased by WT and Cterm GLUT3 chimeras, but not by the ICH and double GLUT3 chimeras (Figure 7c). These experiments implicate the ICH domain of GLUT3 in the activation of PAK signaling. In addition to a role in cytokine receptor endocytosis, the RAS/PAK pathway has also been implicated in macropinocytosis in a wide range of cell types (45). Therefore, we next tested whether GLUT3 might also affect macropinocytosis. Indeed, LysM-Cre *Glut3^fl/fl^* BMDMs showed significantly decreased macropinocytosis, as assessed by FITC-dextran uptake (Figure 7, C and D, and Supplemental Figure 10). Thus, GLUT3 is required for optimal activation of PAK signaling and both IL-4Rα endocytosis and macropinocytosis.

## Discussion

Despite the potential expression of 13 facilitative GLUTs in macrophages, only the functions of GLUT1 and GLUT6 have been investigated. GLUT1 is induced after M1 polarization stimuli, and GLUT1 deletion impaired glycolysis and pentose phosphate pathway activity. However, GLUT1 loss caused variable effects on the inflammatory phenotypes of macrophages in vitro and in vivo, perhaps due to a compensatory increase in oxidative metabolism (19). GLUT6 was also found to be induced by LPS, yet its absence did not cause marked defects in M1 polarization (46, 47). Despite its relatively high expression, the specific functions of GLUT3 in macrophages have not been clarified. Using myeloid cell–specific *Glut1* and *Glut3* deletion mice, we revealed that the 2 proteins have nonredundant roles in specifying macrophage function. Our results are consistent with published *Glut1* overexpression and LysM-Cre *Glut1^fl/fl^* deletion studies that have suggested a role for GLUT1 in glycolytic metabolism and some aspects of M1 macrophage activation (19). We find that LysM-Cre *Glut3^fl/fl^* macrophages show no differences in glucose uptake, glycolysis, pyruvate levels, and ATP levels but do show notable defects in M2 polarization. Glucose flux analyses also reveal no significant differences in glucose utilization by LysM-Cre *Glut3^fl/fl^* macrophages, though a notable limitation of both glucose uptake and flux assays is their dependence on exogenous soluble glucose analogs as markers.

GLUT1 and GLUT3 have previously been reported to exhibit distinct subcellular localizations in both polarized and nonpolarized mammalian cells (48, 49). Previous studies have indicated that GLUT3 localizes primarily to intracellular membranes, rather than exclusively to the plasma membrane of neurons (35). We extended these findings to macrophages with IF and fractionation experiments. We found that GLUT1 localized largely to the plasma membrane, while GLUT3 localized primarily to endosomes. The localization of GLUT3 to intracellular membranes is consistent with its function in endosomal signaling and with the absence of an obvious role in soluble glucose uptake. Notably, we observed that GLUT3-positive endosomes function as signaling endosomes for IL-4/STAT6 signal transduction. Activation of JAK1, which preferentially occurs at the endosome (18), is inhibited by GLUT3 deficiency. Activated p-STAT6 was enriched in the endosomes of WT BMDMs after IL-4 stimulation, but this activation was notably impaired in the endosomes of LysM-Cre *Glut3^fl/fl^* BMDMs. Consistent with the finding that the inhibition of endocytosis with Dynasore could inhibit IL-4/STAT6 activation in WT BMDMs, we found that GLUT3 deficiency inhibited IL-4R endocytosis. Thus, in macrophages, we identify a critical role for GLUT3 in signal transduction, one that is transport independent.

While numerous metabolic enzymes have been demonstrated to possess additional roles in signal transduction (50), examples of nutrient transporters with noncanonical signaling functions are more limited but have been reported. For example, CD36 functions not only as a long-chain fatty acid transporter but also as a scavenger receptor that regulates inflammatory signaling, for both immune and nonimmune cells (51). In addition, GLUT1, but not other GLUT isoforms, has been shown to promote MAPK signaling independent of its function in glucose uptake. RAS regulates cellular metabolism through modulating the transcription of key metabolic enzymes and, at least in one case, through direct binding and modulation of hexokinase 1 (HK1) activity (52, 53). Our study expands the RAS regulatory network further and suggests GLUT3 may regulate RAS signal transduction. Thus, our study provides an additional example of the extensive crosstalk between RAS and metabolic pathways and extends the paradigm that nutrient transporters may also play critical roles in signal transduction.

Our demonstration of a critical role for GLUT3 in coordinating membrane signaling provides context for previous proteomic studies that revealed RAS isoforms (41) as GLUT3-interacting proteins. We confirmed that GLUT3 interacted with RAS in BMDMs by coimmunoprecipitation and also that this interaction required the ICH domain and further demonstrated a direct interaction with KRAS^GTP^ in vitro. The palmitoylation of GLUT1, but not GLUT3, near the ICH motif is necessary for the efficient localization of GLUT1 to the plasma membrane (54). As RAS isoforms are also differentially lipid modified, additional studies will be necessary to determine how lipid modifications of distinct RAS isoforms and GLUT isoforms impact their interactions. While RAS signaling is thought to occur primarily at the plasma membrane, RAS isoforms also localize to endosomes (55, 56). RAS and PAK participate broadly in endocytosis and membrane remodeling, and we found that GLUT3 deficiency reduced phosphorylation of PAK and cofilin in BMDMs. IL-4R subunits are internalized by an actin-dependent endocytic route (18, 38), and our observations of IL-4–activated and GLUT3-dependent changes in p-cofilin are consistent with a role for GLUT3 in coordinating these activities. In summary, we propose that GLUT3 is critical for the function of a signaling complex involving RAS and PAK, which ultimately regulate IL-4R–mediated signal transduction. Previous studies have suggested the coordinated activation of IL-4 signaling and RAC-CDC42-PAK activation (57) or between JAK1/STAT6 and RAS/Erk signaling (58). Our model suggests that crosstalk between these pathways could occur through GLUT3 at the level of RTK endocytosis and activation. Additional studies that specifically address the links between IL-4 and other RTKs and the RAS/PAK pathway are necessary to confirm and extend our findings.

While we specifically delineated an upstream role for GLUT3 in IL-4/STAT6 signaling in macrophages, it is likely that its role in promoting signal transduction is more broadly conserved. Many RTKs, and cytokine receptors in particular, require endocytosis to endosomes to promote signaling. However, not all cytokines require endocytosis and endosomal enrichment for signal transduction. Specifically, IFN-γ signaling occurs efficiently at the plasma membrane (59), perhaps explaining why M1 polarization stimuli may not be abrogated by loss of GLUT3. Moreover, GLUT3 does not appear to be required for all forms of endocytosis, as TLR4 endocytosis was not affected by GLUT3 deficiency. A more detailed catalog of the specific cytokines and signaling pathways that require GLUT3 for optimal signal transduction will require further investigation.

Overall, our findings suggest that GLUT3 functions in membrane dynamics and signal transduction. Like myeloid cells, many of the cell types in which GLUT3 is highly expressed — neurons, melanocytes, Langerhans cells, platelets, and others — share the feature of having extensive, compartmentalized endomembrane systems. We speculate that GLUT3 is important for the compartmentalization and maintenance of these endomembrane complexes. Our studies in PBMCs, BMDMs, and macrophage cell lines reveal a function for GLUT3 in M2 polarization and signaling. Human disease tissues and mouse models of wound healing and atopic dermatitis confirm the critical role of GLUT3 in signaling in vivo and demonstrate the biological importance of this specific GLUT. It will be important to determine whether GLUT3’s role in RAS binding and signal transduction is conserved in other cell types and how the complex contributes to other disease states, including cancer.

## Methods

### Animal studies.

All efforts were made to follow the Replacement, Refinement and Reduction guidelines. *Slc2a1^fl/fl^* (*Glut1^fl/fl^*) and *Slc2a3^fl/fl^* (*Glut3^fl/fl^*) mice were supplied in-house. B6.129P2-Lyz2^tCAM(cre)Ifo^/J (LysM-Cre) mice were obtained from The Jackson Laboratory (stock number 004781). For the calcipotriol-induced (MC903-induced) atopic dermatitis model, 1.125 nmol calcipotriol in ethanol was applied to the right ear and the shaved back of mice for 13 days. Wound healing assays were completed as previously described (12). Briefly, an excisional wound was generated on the shaved back skin of mice with a 3 mm punch biopsy. Wounds were splinted with a silicone ring and covered by antibiotic ointment and Tegaderm. Wound size was measured every 2 days, at which time Tegaderm and antibiotic ointment were replaced.

### Preparation of mouse BMDMs.

Bone marrow was harvested from age-matched male WT (LysM-Cre^–/–^;*Glut1^fl/fl^* or LysM-Cre^–/–^; *Glut3^fl/fl^*), LysM-Cre^+/–^;*Glut1^fl/fl^* and LysM-Cre^+/–^;*Glut3^fl/fl^* as previously described, with minor modifications (60). BMDMs were generated by culturing marrow cells in poly-L-lysine–coated culture plates for 7 days with 50 ng/mL M-CSF in RPMI 1640 (Sigma-Aldrich, R8758) supplemented with 20% FBS, 1× GlutaMax (Thermo Fisher Scientific, 35050061) and 1× Antibiotic-Antimycotic solution (Thermo Fisher Scientific, 15240062). BMDMs were activated using 100 ng/mL LPS and 50 ng/mL IFN-γ (for M1) or 10 ng/mL IL-4 (for M2) for 24 hours.

### Preparation of primary human PBMC-derived macrophages.

Primary human CD14^+^ PBMCs (STEMCELL Technologies) were purchased and cultured as previously described (61). Briefly, cells were cultured in RPMI 1640 media supplemented with 10% FBS and 20 ng/mL recombinant human M-CSF. The next day, 1 mL of media was carefully removed from each well and replaced with fresh RPMI supplemented with 10% FBS and 20 ng/mL recombinant human M-CSF. On day 4, nonadherent cells were removed by washing once and cultured with fresh RPMI supplemented with 10% FBS and 5 ng/mL recombinant human M-CSF. On day 5, the cells were activated with 20 ng/mL recombinant human IFN-γ and 5 ng/mL LPS (for M1) or 20 ng/mL IL-4 (for M2) for 24 hours.

### Cell lines and culture.

Human macrophage THP-1 cells (ATCC) were cultured in RPMI 1640 supplemented with 10% FBS and 1× Antibiotic-Antimycotic solution. THP-1 monocytes are differentiated into macrophages by 36-hour incubation with 20 nM phorbol 12-myristate 13-acetate (PMA; Sigma-Aldrich, P8139). Murine macrophage Raw 274.7 cells (ATCC) were cultured in DMEM (Sigma-Aldrich, D5796) supplemented with 10% FBS and 1× Antibiotic-Antimycotic solution. All cells were grown at 37°C with 5% CO_2_. For serum starvation and stimulation, cells were incubated in DMEM without FBS for 16 hours, and then cells were stimulated with 20% FBS for 10 minutes.

### [^3^H]2-DG uptake, pyruvate, ATP, and extracellular lactate assays.

2-DG uptake was measured as previously described (62). Briefly, BMDMs from WT, LysM-Cre^–/–^*;Glut1^fl/fl^* or LysM-Cre^–/–^;*Glut3^fl/fl^*, LysM-Cre^+/–^;*Glut1^fl/fl^*, and LysM-Cre^+/–^;*Glut3^fl/fl^* mice were seeded in triplicate into 12-well plates overnight. The cells were washed twice with PBS and incubated in basic serum-free DMEM for 2 hours. Uptake was initiated by addition of 1 μCi [^3^H]2-DG (25–30 Ci/mmol, PerkinElmer, NET549) and 0.1 mM unlabeled 2-DG (Sigma-Aldrich, D8375) to each well for 10 minutes. Transport activity was terminated by rapid removal of the uptake medium and subsequently washing 3 times with cold PBS with 25 mM glucose (Sigma-Aldrich, G7528). Cells were lysed with 0.5 mL of 0.5 M NaOH (Thermo Fisher Scientific, SS255-1) and neutralized with 0.5 mL of 0.5 M HCl (Sigma-Aldrich, 320331), which was added and mixed well. Two hundred and fifty microliters of the lysate was transferred to a scintillation vial containing scintillation solution, and the sample was analyzed by liquid scintillation counting. Protein concentrations were determined through BCA assays (Thermo Fisher Scientific, 23227). To allow for normalization across multiple BMDM preparations, 2-DG after M1 or M2 polarization was measured and then normalized to uptake from the same BMDM preparation in the unstimulated (M0) state. Intracellular pyruvate and ATP levels were quantified using commercial assay kits (Sigma-Aldrich, MAK071 and Abcam, ab83355) according to the manufacturers’ instructions. For extracellular lactate measurements, BMDMs were cultured and activated with the indicated polarization stimuli for 24 hours as described above. One milliliter of culture media was collected, spun down to remove any cells, and the concentration of lactate was measured using an automated electrochemical analyzer (Bioprofile Basic-4 analyzer, NOVA).

### ^13^C_6_-glucose isotopic tracing assay.

BMDMs at approximately 90% confluence in 6-well plates were switched to glucose-free RPMI (Gibco, 11-879-020) containing 10% dialyzed FBS and 10 mM ^13^C_6_-glucose (Cambridge Isotope Laboratories, CLM-1396-PK) for 3 hours (63). Metabolites were extracted using 80% ice-cold methanol and dried down in a SpeedVac concentrator. The dried samples were either resuspended in 80% acetonitrile for liquid chromatography–tandem mass spectrometry (LC-MS/MS) or derivatized for gas chromatography (GC-MS/MS). For isotopologue analysis by LC, the dried metabolites from ^13^C_6_-glucose isotope tracing were resuspended in 100 μL of 80% acetonitrile and run on a Q-Exactive mass spectrometer (Thermo Fisher Scientific) coupled to a Prominence UPLC system (Shimadzu) with Amide XBridge HILIC chromatography. The peak areas of metabolites were integrated using TraceFinder 5.1 (Thermo Fisher Scientific). Glucose peaks were obtained using the Q-Exactive MS. For isotopologue analysis by GC, the dried metabolites were resuspended in 20 μL of pyridine-containing methoxyamine (10 mg/mL). Samples were heated at 70°C for 15 minutes, followed by addition of 50 μL of *N*-(*tert*-butyldimethylsilyl)-*N*-methyltrifluoroacetamide (Sigma-Aldrich, 77626). Samples were heated at 70°C for another 70 minutes. One microliter of each sample was injected and analyzed on an Agilent 7890A gas chromatograph coupled to an Agilent 5975C mass selective detector. The peaks for pyruvate and lactate were obtained using GC.

### Single-cell RNA-seq analysis.

Atopic dermatitis and healthy control cutaneous immune single-cell RNA-seq (scRNA-seq) data (EGA EGAS00001005271) were analyzed as previously described (22). Diabetic and nondiabetic foot ulcer scRNA-seq data were obtained from the NCBI Gene Expression Omnibus (GEO) database (GSE165816). Diabetic and nondiabetic foot ulcer samples underwent quality control filtering after a Seurat object for each sample was made. Cells with less than 50% of mitochondrial counts, cells expressing more than 200 genes, and genes uniquely expressed in more than 3 cells were retained. Samples were then merged and “NormalizeData,” “FindVariableFeatures,” “ScaleData,” and “RunPCA” functions were applied. Integration and batch effect correction step was performed using the “RunHarmony” function from the harmony R package (64). Then, the Uniform Manifold Approximation and Projection (UMAP) method was applied to reduce the dimension of the data by setting the “dims” parameter to 23, and cells were clustered with resolution of 0.4. M1 and M2 macrophage clusters were identified by expression of *IL1B* and *CD163*, respectively. Differential expression analysis for the genes *SLC2A1* (GLUT1) and *SLC2A3* (GLUT3) between disease groups on a per-cluster basis was performed with Seurat using MAST differential expression analysis.

### GLUT3 shRNAs, GLUT3 expression alleles, and lentiviral transductions.

*GLUT3* shRNA sequences were designed for the pLKO.1 vector using the TRC shRNA Design Process (https://portals.broadinstitute.org/gpp/public/resources/rules). Forward and reverse oligonucleotides were annealed in NEB buffer 2 (New England Biolabs), heated at 95°C for 10 minutes, slowly cool to room temperature, and then ligated into the pLKO.1 vector (Addgene, 10878) using *Age*I/*Eco*RI. The constructs were confirmed by Sanger sequencing. An amino-terminal 3×FLAG epitope–tagged human GLUT3 was generated by PCR (Addgene, 72877) (Supplemental Table 1). Missense mutants (N32S, G312S, N315T, R331W) and shGLUT3-resistant GLUT3 alleles (sh1sh3 resist) were designed and synthesized as DNA fragments by Integrated DNA Technology (Supplemental Table 1). After PCR amplification, GLUT3 missense mutants, the GLUT3 sh1sh3 resistant mutant, and double mutants were cloned by restriction digestion. shRNA-resistant chimeric mutants of FLAG-tagged GLUT3 alleles were generated using NEBuilder HiFi DNA Assembly Cloning Kit (New England Biolabs, E5520) according to the manufacturer’s protocol. Vector (WT GLUT3) and inserts (GLUT3 ICH, GLUT3 Cterm) were amplified by PCR using the indicated the primer sets (Supplemental Table 1). PCR fragments were assembled after incubation at 50°C for 30 minutes with the NEBuilder HiFi DNA Assembly Master Mix in the kit. All constructs were confirmed by Sanger sequencing.

For generating lentiviruses, LentiX-293T cells (Clontech, 632180) were seeded at approximately 60% confluence in antibiotic-free media 12 to 16 hours before transfection. shRNA (4.5 μg) or expression plasmid (4.5 μg), 2.5 μg of pMD2.G (Addgene, 12259), and 4.5 μg of psPAX2 (Addgene, 12260) were cotransfected into LentiX-293T cells using Lipofectamine 3000 (Thermo Fisher Scientific, L3000015) according to the manufacturer’s protocol. Viruses were collected after 48 and 72 hours of transfection. For lentiviral transduction, THP-1 cells were seeded into 6-well plates at 70% confluence. Viral supernatant was then added to the cells with polybrene at a concentration of 8 μg/mL. Cells were selected with puromycin antibiotic at a concentration of 2 μg/mL and hygromycin at a concentration of 100 μg/mL. For double transductions (GLUT3 allele + shGLUT3), unmodified THP-1 cells were serially transduced first with the GLUT3 expression plasmid by puromycin selection followed by the shGLUT3 allele by hygromycin selection.

### siRNA interference.

siRNA targeting mouse *Slc2a3* (GLUT3) was synthesized by Sigma-Aldrich. Cells were transfected with 100 nM siRNA using Lipofectamine RNAiMAX reagent (Thermo Fisher Scientific, 13778150) according to the manufacturer’s protocol.

### RNA extraction and qRT–PCR.

RNA was extracted form cells or tissue using an RNeasy Mini Kit (Qiagen, 74106) and reverse transcribed to cDNAs using an Iscript cDNA Synthesis Kit (Bio-Rad, 1708891) according to the manufacturer’s protocol. qRT-PCR analyses were performed using the cDNAs from the reverse transcription reactions, gene-specific primers, and PowerUp SYBR Green (Applied Biosystems, A25779). All primers for qRT-PCR are listed in Supplemental Tables 1 and 2.

### Isolation of plasma membranes and endosomes.

Cells were fractionated into plasma membranes and endosomes using fractionation kits (Invent Biotechnologies, SM-005 and ED-028) according to the manufacturer’s protocol. Briefly, cells were lysed with the supplied buffer and intact nuclei and unruptured cells were removed by the filter cartridge and brief centrifugation. The supernatant was incubated with the supplied precipitation buffer to isolate and enrich the plasma membranes or endosomes.

### Immunoblotting and immunoprecipitation.

For STAT6 signaling experiments, cells were stimulated with 20 ng/mL IL-4 for 30 minutes. Dynasore (200 μM; Sigma-Aldrich, D7693) was applied for 30 minutes before IL-4 treatment. Cells were pretreated with G3iA for 10 minutes before IL-4 treatment. After stimulation, cells were lysed with Cell Lysis Buffer (Cell Signaling Technology, 9803) and whole-cell lysates (WCLs) were subjected to Laemmli Sample Buffer (Bio-Rad, 1610747) and sodium dodecyl sulfate–polyacrylamide gel electrophoresis (SDS-PAGE). The separated proteins were transferred to a nitrocellulose membrane and incubated with primary antibodies against GLUT3 (Abcam, ab191071, EPR10508(N), or ab15311), GLUT1 (MilliporeSigma, 07-1401), p-STAT1 (Y701) (Cell Signaling Technology, 7649, D4A7), STAT1 (Cell Signaling Technology, 14994, D1K9Y), p-STAT6 (Y641) (Cell Signaling Technology, 9361), STAT6 (Cell Signaling Technology, 5397, D3H4), p-JAK1(Y1034/1035) (Cell Signaling Technology, 3331), JAK1 (Cell Signaling Technology, 3332), Na^+^/K^+^-ATPase (Cell Signaling Technology, 3010), EEA1 (Cell Signaling Technology, 48453, E9Q6G), IL-4Rα (OriGene Technologies, AP20570PU-N), γ_c_ chain (R&D Systems, AF284), p-PAK (PAK1[T423]/PAK2[T402]) (Cell Signaling Technology, 2601), PAK1 (Cell Signaling Technology, 2602), pan-RAS (Cell Signaling Technology, 8955, D2C1), GST (Santa Cruz Biotechnology, sc-138, B-14), and Hsp90 (Cell Signaling Technology, 4877). After incubating with horseradish peroxidase–conjugated secondary antibodies, antigens were visualized using Western Lightning Plus-ECL (PerkinElmer, 50-904-9323).

For immunoprecipitation, cells were lysed in buffer containing 20 mM Tris-HCl (pH 7.4), 137 mM NaCl, 1 mM MgCl_2_, and 1 mM CaCl_2_. WCLs were incubated with anti-GLUT3 antibody overnight and then with Protein A/G PLUS-Agarose bead slurry (Santa Cruz Biotechnology, sc-2003) for 3 hours. Immunoprecipitates were analyzed by immunoblotting.

### GST binding assay.

Raf-1 GST RBD 1–149 was a gift from Channing Der (Addgene plasmid 13338) (65). The pGEX-2T backbone was used for the GST control. GST GLUT1 ICH was generated as previously described (55). Fragments corresponding to the GLUT3 ICH and Cterm domains were generated by PCR using the indicated primers (Supplemental Table 1) and cloned into pGEX-2T as *Bam*HI/*Eco*RI fragments. GST expression plasmids were transformed into BL21 cells, grown in Luria broth (LB) to OD_600_ of 0.6, and induced with 0.5 mM isopropyl β-D-1-thiogalactopyranoside for 16 hours at 20°C. Bacteria were pelleted, washed in PBS, and resuspended in GST wash buffer (50 mM Tris pH 8.0, 150 mM NaCl, 1 mM DTT, protease inhibitor [Pierce, A329630]). Bacteria were lysed by sonication, pelleted by centrifugation at 10,000*g*, and lysates incubated with 0.2 mL glutathione-agarose columns (Pierce, PI16103) in duplicate for 60 minutes at 4°C. Full-length KRAS^WT^ (amino acids 1–188) was prepared as previously described (66, 67). Nucleotide exchange to GDP or 5′-guanylyl-imidodiphosphate (GMP-PNP) was done by incubating N-terminal His-tagged tobacco etch virus (TEV)–KRAS^WT^ at a concentration of 2.5 mg/mL with GDP or GMP-PNP at 2.5 mM in a total volume of 400 μL of loading buffer (20 mM Tris pH 7.5, 50 mM NaCl, 200 mM [NH_4_]_2_SO_4_, 10 mM EDTA) for 2 hours at 25°C. After incubation, the nucleotide-loaded KRAS proteins were exchanged into assay buffer (20 mM Tris pH 7.5, 150 mM NaCl, 5 mM MgCl_2_, 1 mM EDTA) to terminate the nucleotide loading reaction using Zeba Spin Desalting Columns (Thermo Fisher Scientific, 89890). For the binding assay, after washing 3 times with GST wash buffer, columns were incubated with 80 μg of GDP- or GMP-PNP–loaded KRAS^WT^ protein in binding buffer (1× PBS [pH 7.4], 0.5% gelatin, 0.05% Tween 20) for 2 hours at 4°C. After washing 3 times with binding buffer, columns were treated with 200 μL elution buffer (50 mM Tris pH 8.0, 10 mM glutathione [reduced], 150 mM NaCl, 1mM DTT). Eluted proteins were normalized to levels of GST fusion protein by Coomassie staining and Western blotting.

### Immunofluorescent staining.

For immunofluorescent staining of cells, cells were fixed with 4% paraformaldehyde in PBS for 10 minutes, permeabilized with 0.1% Triton X-100 for 10 minutes, and blocked with blocking solution (3% BSA in PBS) for 1 hour. Cells were then incubated with primary antibodies against GLUT1 (MilliporeSigma, 07-1401), GLUT3 (Santa Cruz Biotechnology, sc-30107), and EEA1 (Cell Signaling Technology, 48453) in blocking buffer overnight at 4°C, followed by 1 hour of incubation with fluorescent dye–labeled secondary antibodies (Thermo Fisher Scientific, A11005 or A11008). After mounting with Cytoseal 60 (Thermo Fisher Scientific, 23244257), confocal images were captured on an LSM 780 confocal microscope (Zeiss).

For immunofluorescent staining of tissues, tissues were fixed in 4% paraformaldehyde and embedded in paraffin. Sections (5 μm) were deparaffinized, heat retrieved at 95°C for 30 minutes with citrate buffer (Thermo Fisher Scientific, AP-9003-125), permeabilized with 0.1% Triton X-100 for 10 minutes, and blocked with 5% goat serum and 0.2% BSA in PBS. Tissues were then incubated with primary antibodies against Arg1 (Cell Signaling Technology, 93668), F4/80 (Thermo Fisher Scientific, MA1-91124), GLUT3 (Proteintech, 20403-1-AP), and CD68 (Thermo Fisher Scientific, PIMA513324) overnight at 4°C, followed by 1 hour of incubation with fluorescent dye–labeled secondary antibodies (Thermo Fisher Scientific, A11005 or A11007). After mounting with ProLong Gold antifade reagent with DAPI (Thermo Fisher Scientific, P36935), confocal images were captured on an LSM 780 confocal microscope (Zeiss).

### FITC-dextran (macropinocytosis) assay.

The FITC-dextran uptake assay was performed as previously described (68). BMDMs were cultured overnight in complete RPMI medium (20% FBS, 1× Glutamax, and 1× Antibiotic-Antimycotic solution) in the absence of M-CSF to render macrophages quiescent. The following morning, the BMDMs were incubated with 500 μg/mL FITC-conjugated 70 kDa dextran (Invitrogen, D1823) in the presence of 50 ng/mL M-CSF in serum-free RPMI for 15 minutes at 37°C. Then, cells were placed on ice for 5 minutes to inhibit uptake of FITC-dextran and cells were washed twice with cold PBS. For a negative control, BMDMs were incubated with FITC-dextran on ice.

### Statistics.

The sample sizes and statistical tests, including corrections for multiple comparisons, that were used for each experiment are described in the relevant figure legend. A *P* value of 0.05 or less was considered significant: *P ≤ 0.05, **P ≤ 0.01, ***P ≤ 0.001, ****P ≤ 0.0001. All *t* tests were 2-tailed. ANOVA tests were completed as indicated in the figure legends.

### Study approval.

Animal studies were performed in accordance with the recommendations in the NIH *Guide for the Care and Use of Laboratory Animals* (National Academies Press, 2011). All animal studies were conducted in accordance with institutional guidelines and was approved by the Institutional Animal Care and Use Committee (IACUC), animal protocol number 2015–101166 of the UT Southwestern (UTSW). All efforts were made to follow the Replacement, Refinement and Reduction guidelines. Human studies were approved by the UTSW institutional review board (IRB) (STU-072018-067). Only deidentified, anonymized human tissues were used in the study. Excess FFPE sections from skin biopsies obtained in the course of standard- of-care therapies from patients with atopic dermatitis or healing wounds were used for IF analyses.

### Data availability.

All data supporting the findings of this study are available within the paper, the Supporting Data Values file, and complete unedited blots are in the supplemental material.

## Author contributions

DMY, JZ, EEL, DK, KDW, JYC, and RCW designed the study. DMY, JZ, EEL, DK, RM, EKR, ZZ, CH, and RCW conducted experiments. DMY, JZ, DK, AEK, JYC, GH, RJC, JBC, and RCW analyzed data. ZZ and KDW provided reagents. EDA provided *Slc2a1^fl/fl^* (*Glut1^fl/fl^*) and *Slc2a3^fl/fl^* (*Glut3^fl/fl^*) mice. JYC provided G3iA. DMY and RCW prepared figures and wrote the manuscript. RCW acquired funding.

## Supplementary Material

Supplemental data

Supporting data values

## Version 1 09/18/2023

In-Press Preview

## Version 2 11/01/2023

Electronic publication
