# Supplementary material for: GLUT3 promotes macrophage signaling and function via RAS-mediated endocytosis in atopic dermatitis and wound healing
Source: J Clin Invest. 2023 Nov 1;133(21):e170706. doi: 10.1172/JCI170706 (PMC10617774; doi:10.1172/JCI170706)

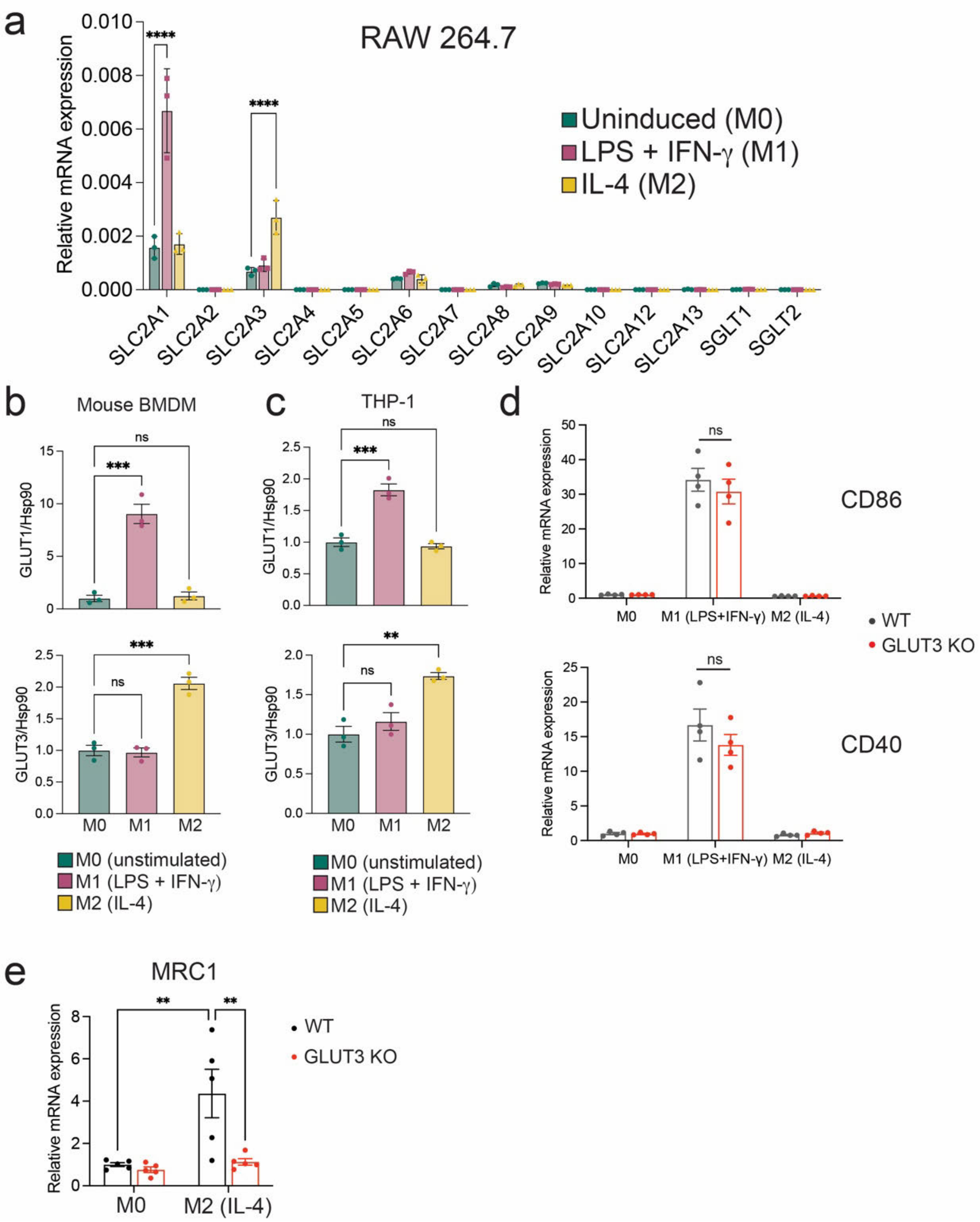

Supplementary Figure 1. Expression of glucose transporter isoforms and effect of GLUT1 and GLUT3 KO. (a) Expression of glucose transporters in Raw 264.7 cells. mRNA expression levels of GLUT and SGLT transporter isoforms in Raw 264.7 cells in unstimulated macrophages (white) and after treatment with M1 (red) or M2 (blue) polarization stimuli for 24 hours. **(b)** Quantitation of Western blots assessing expression of GLUT1 and GLUT3 with the indicated polarization stimuli in BMDM (b) and THP-1 (c). Mean of GLUT1 or GLUT3 relative to HSP90 levels from quantification of WB (n=3 biological replicates). **(d)** Effect of GLUT1 KO on the mRNA levels of CD86 and CD40, cell surface markers of M1 polarization (n=4 biological replicates). **(e)** Effect of GLUT3 KO on the mRNA levels of MRC1, a cell surface marker of M2 polarization (n=5 biological replicates). Data shown as mean ± SEM. P values were calculated by two-way ANOVA with Tukey's test. *P≤0.05, **P≤0.01, ***P≤0.001, ****P≤0.0001.

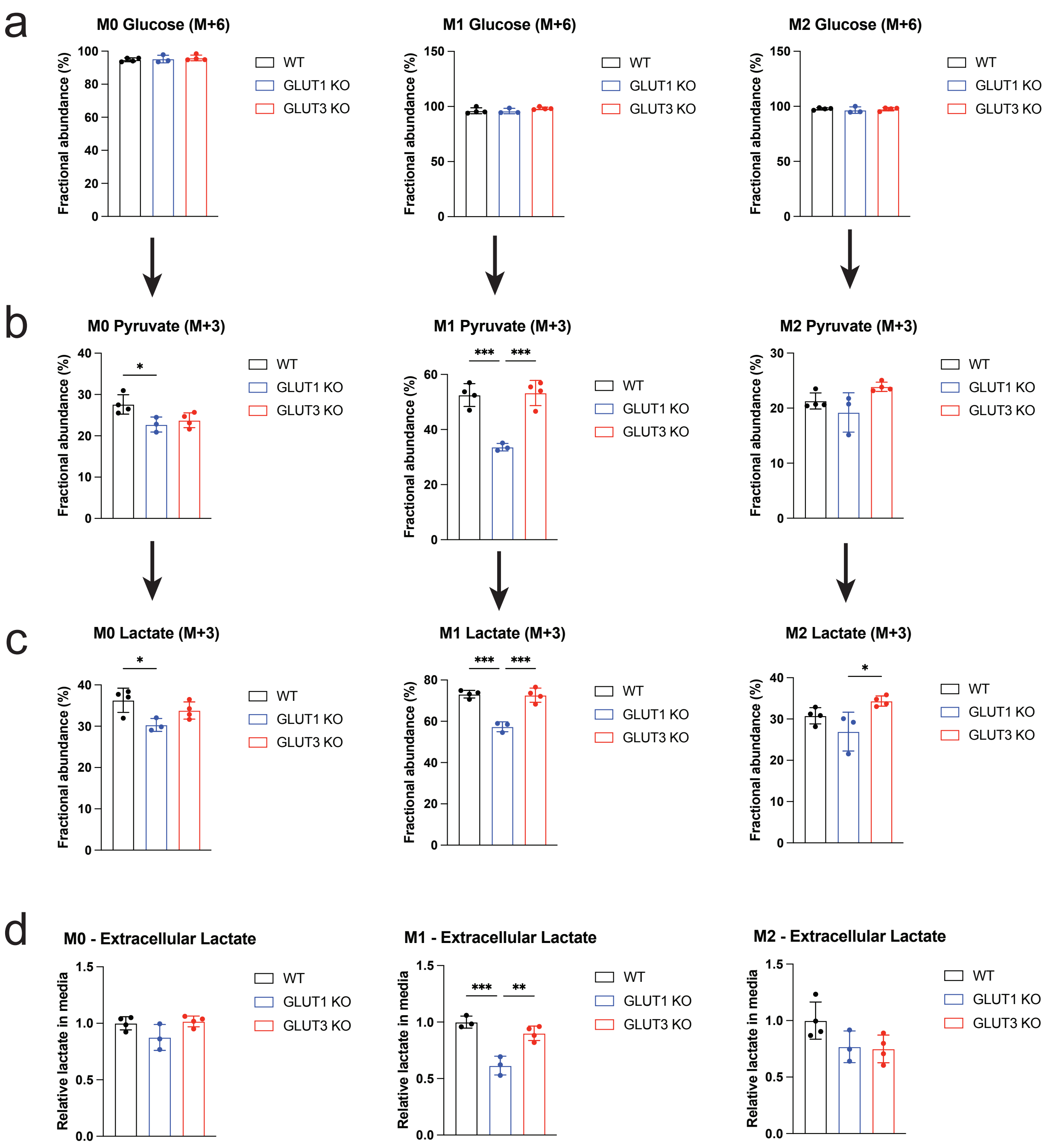

Supplementary Figure 2. Glucose metabolism in GLUT1 and GLUT3 KO BMDM. Fractional abundance of indicated $^{13}C_6$ isotopologue—M+6 glucose **(a)**, M+3 pyruvate **(b)**, and M+3 lactate **(c)** in BMDM treated with the indicated polarization stimuli. **(d)** Extracellular lactate measured by Nova Bio-analyzer in BMDM treated with the indicated polarization stimuli.

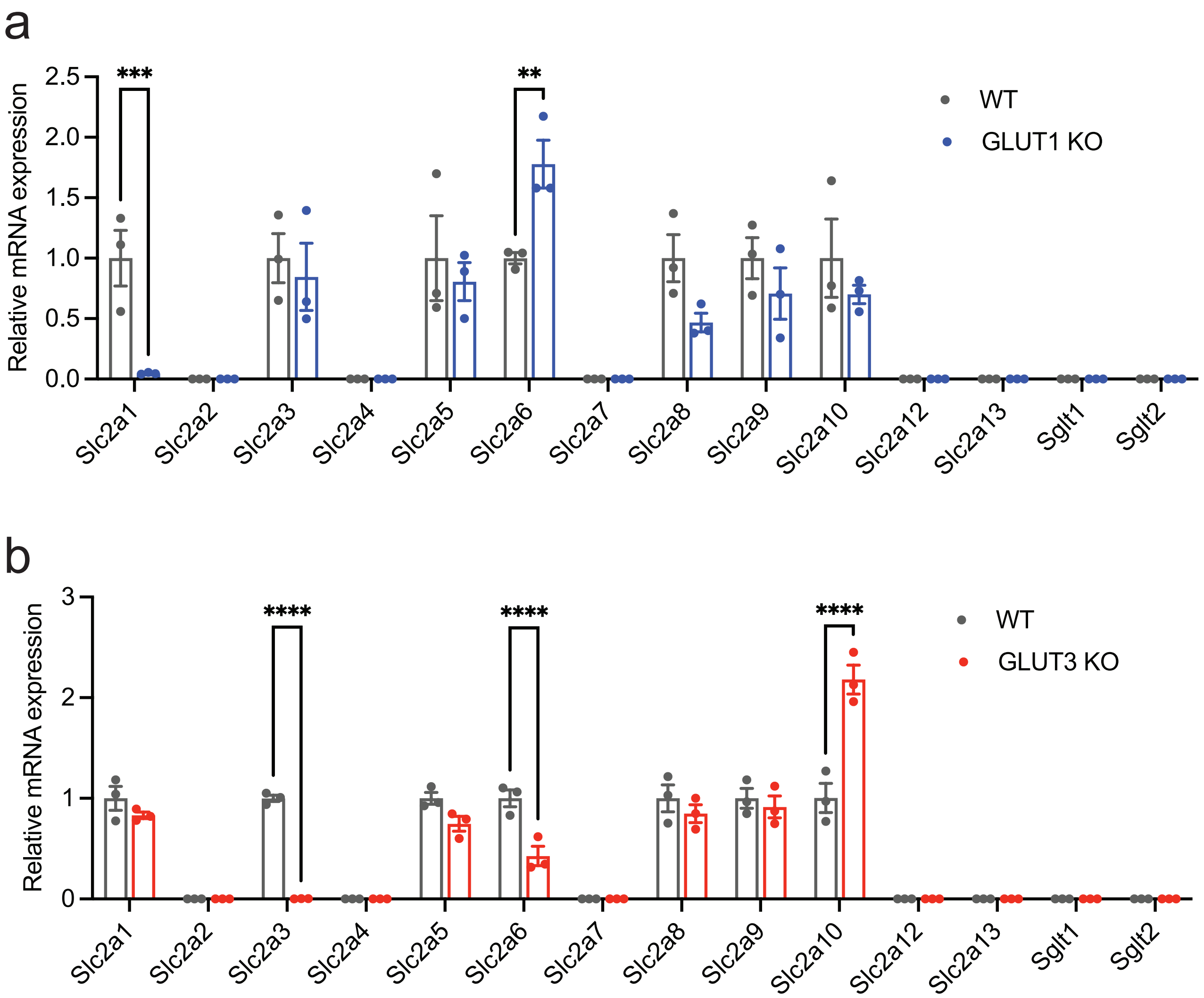

Supplementary Figure 3. Glucose transporter isoform expression after GLUT1 and GLUT3 KO. mRNA expression levels of GLUT and SGLT transporter isoforms in GLUT1 KO (a) and GLUT3 KO (b) BMDMs unstimulated macrophages. Expression normalized to β-actin (ACTB) expression (n=3 biological replicates). P values were calculated by two-way ANOVA with Sidak's test (a-b). $^{**}P \leq 0.01$, $^{***}P \leq 0.001$, $^{****}P \leq 0.0001$.

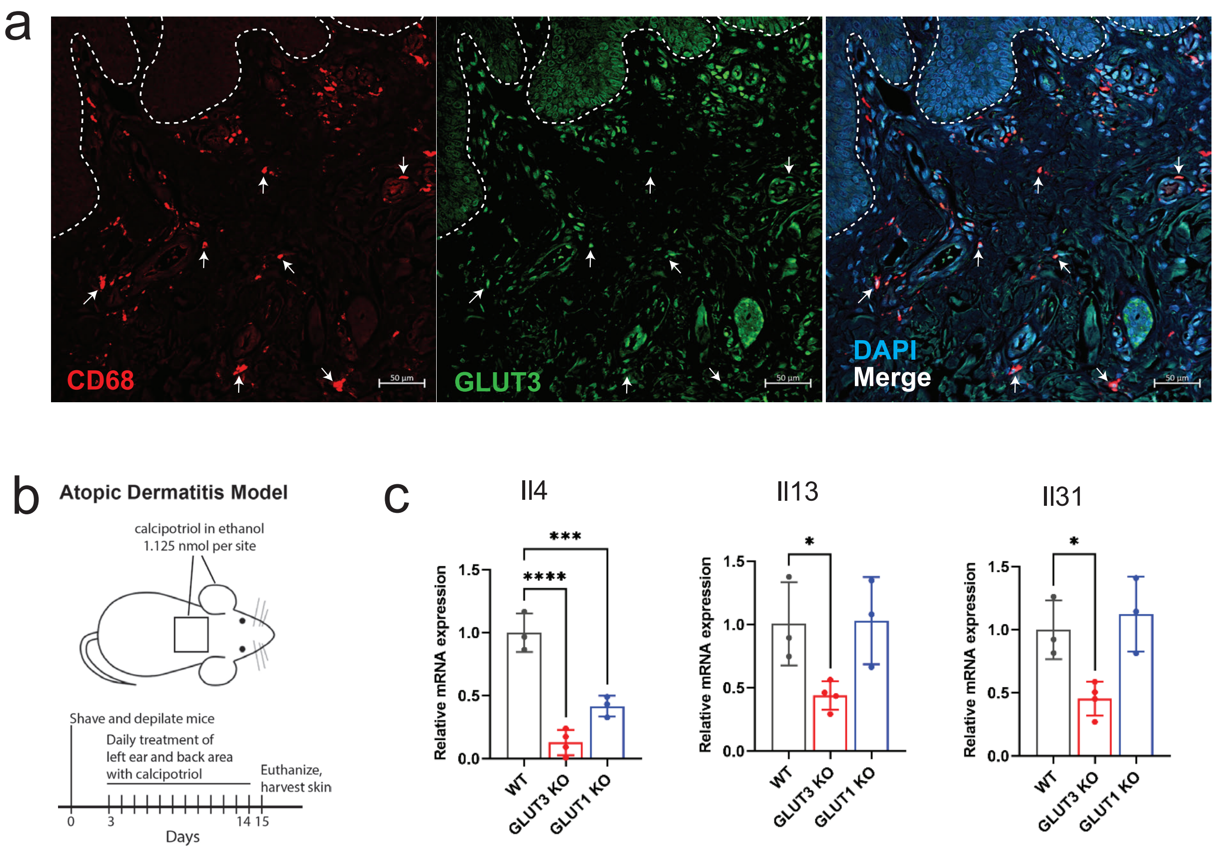

Supplementary Figure 4. The role of GLUT3 in atopic dermatitis and calcipotriol induced inflammation. (a) Representative immunofluorescence stains of a patient biopsy specimen of atopic dermatitis with CD68 (red), GLUT3 (green), and DAPI (blue). Arrows indicate cells expressing both CD68 and GLUT3 in dermal infiltrate. Dotted line indicates dermo-epidermal junction. Scale bar = 50 μm. (Supplementary Fig. 5a). **(b)** Scheme for calcipotriol (MC903) induced dermatitis. Calcipotriol (1.125 nmol) in ethanol was applied to the left ear and shaved back of the indicated mice for 13 days. **(c)** mRNA expression levels of Th2 cytokines (*Il4*, *Il13*, and *Il31*) in calcipotriol-treated ear in WT (n=3), GLUT3 KO (n=4), and GLUT1 KO (n=3) mice. Data shown as mean ± SEM. P values were calculated by one-way ANOVA with Dunnett's test. *P ≤0.05, **P≤0.01, ***P≤0.001, ****P≤0.0001.

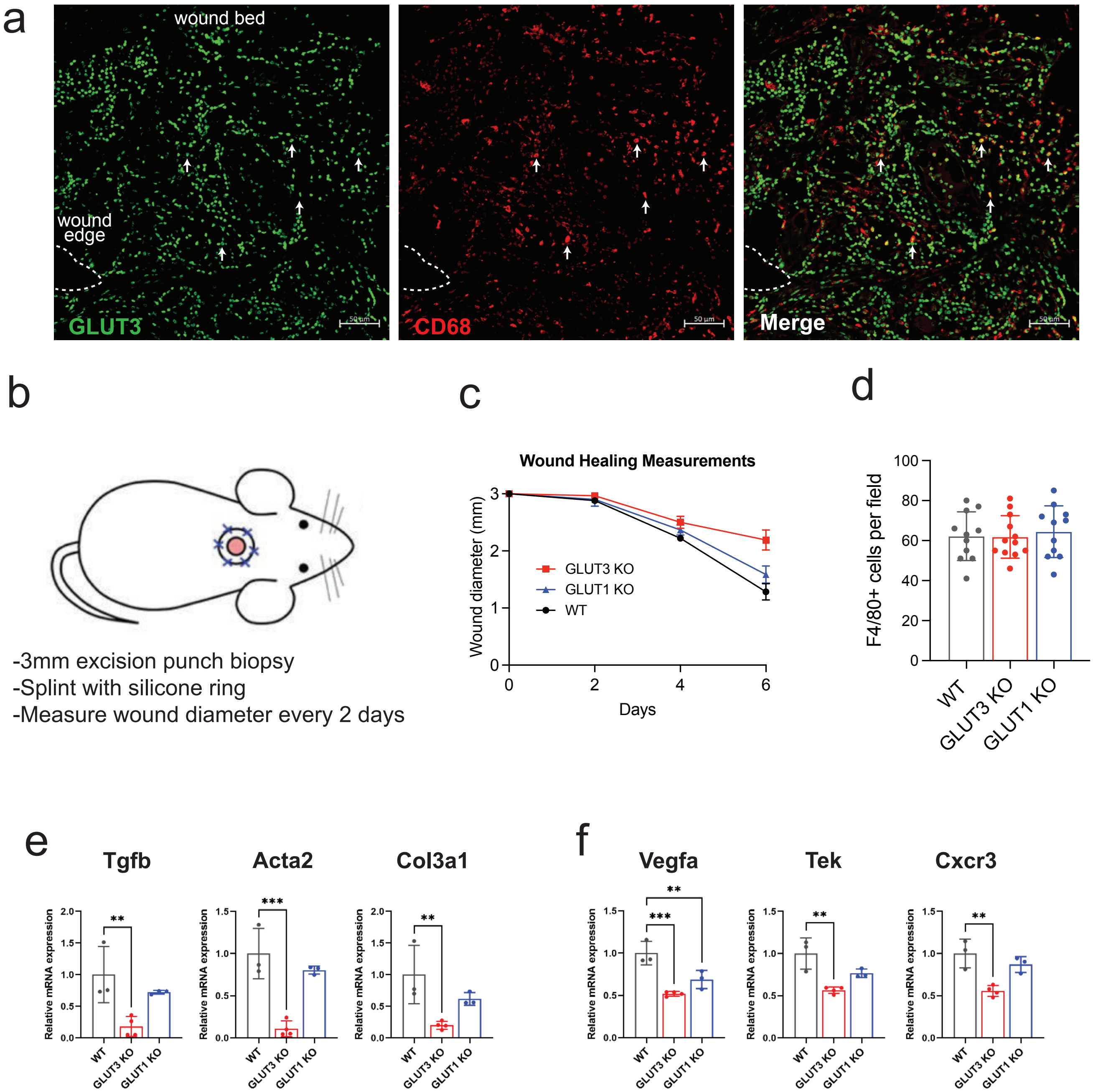

Supplementary Figure 5. Wound healing in GLUT1 and GLUT3 KO mice. (a) Representative immunofluorescence stains of a patient biopsy specimen of a healing wound with CD68 (red), GLUT3 (green). Arrows indicate cells expressing both CD68 and GLUT3 in the wound bed. Dotted line indicates wound edge. Scale bar = 50 μm. (Supplementary Fig. 5a). **(b)** Scheme for splinted wound healing model. Wounds on the shaved back of WT, GLUT1, and GLUT3 KO mice were generated by 3.0 mm punch biopsy, splinted, and wound healing was measured. **(c)** Splinted wounds were measured on Day 2, 4, and 6. Data shown as mean ± SEM. WT (n=12), GLUT3 KO (n=12) and GLUT1 KO (n=7). **(d)** Quantitation of F4/80+ cells (red) per high power field at 6 days after injury. **(e-f)** mRNA expression levels of tissue-remodeling related markers (*Tgfb*, *Acta2* and *Col3a1*) (h) and angiogenesis markers (Vegfa, Tek and Cxcr3). Data shown as mean ± SEM. P values were calculated by one-way ANOVA with Dunnett's test. *P≤0.05, **P≤0.01, ***P≤0.001, ****P≤0.0001.

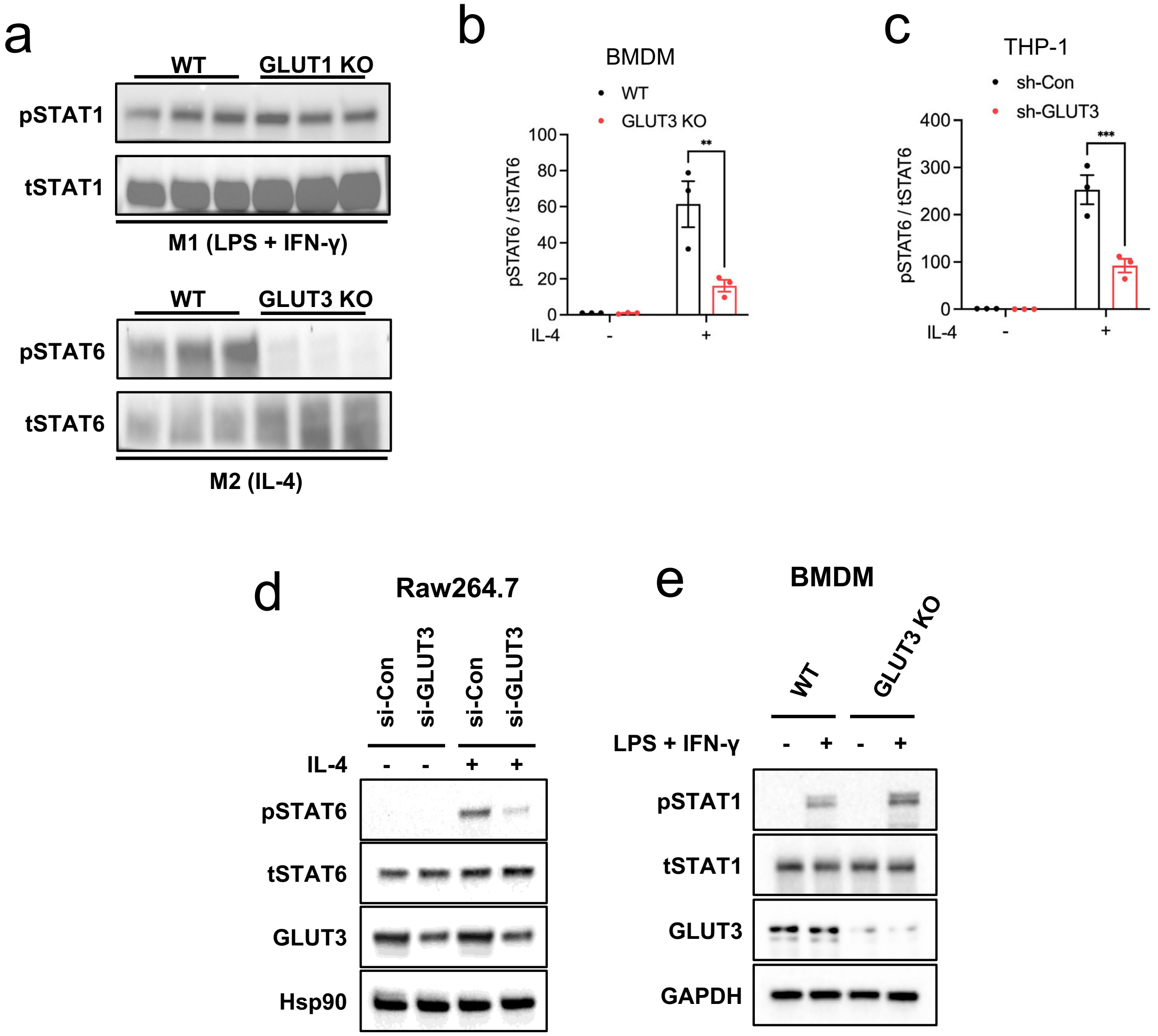

Supplementary Figure 6. STAT activation after inhibition of GLUT1 and GLUT3. (a) Western blot analysis of the expression of phospho-STAT1 (Y701) and total STAT1 in WT and GLUT1 KO BMDMs after LPS and IFNγ stimulation (upper panel). The expression of phospho-STAT6 (Y641) and total STAT6 in WT and GLUT3 KO BMDMs after IL-4 stimulation (lower panel). **(b)** Quantification of pSTAT6/tSTAT6 levels from WB of WT or GLUT3 KO BMDM -/+ IL-4 stimulation (30 min) (n=3 biological replicates). **(c)** Quantification of pSTAT6/tSTAT6 levels from WB of control or GLUT3 shRNA transduced THP-1 -/+ IL-4 stimulation (30 min) (n=3 biological replicates). **(d)** Western blot for pSTAT6 and tSTAT6 +/- IL-4 activation (30 minutes) after siRNA knockdown of endogenous GLUT3 in Raw264.7 cells. **(e)** Western blot for phospho-STAT1 (pSTAT1) and total STAT1 (tSTAT1) +/- LPS and IFN-γ activation (30 minutes) in WT and GLUT3 KO BMDMs. P values were calculated by one-way ANOVA with Dunnett's test. *P≤0.05, **P≤0.01, ***P≤0.001, ****P≤0.0001.

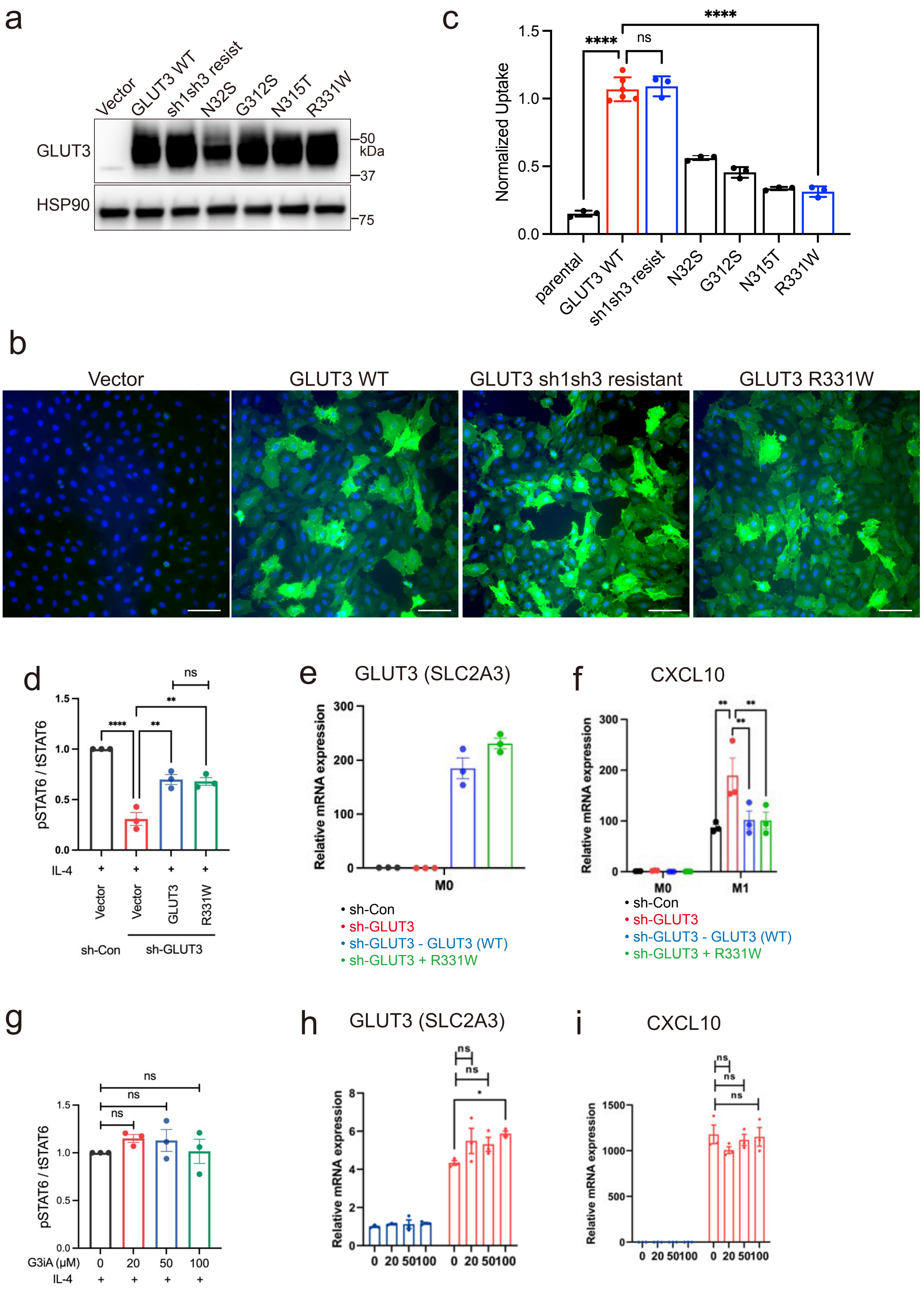
a
Vector
GLUT3 WT
sh1sh3 resist
N32S
G312S
N315T
R331W
GLUT3
HSP90
50 kDa
37
75
c
Normalized Uptake
parental
GLUT3 WT
sh1sh3 resist
N32S
G312S
N315T
R331W
ns
****
b
Vector
GLUT3 WT
GLUT3 sh1sh3 resistant
GLUT3 R331W
d
pSTAT6 / tSTAT6
IL-4
sh-Con
sh-GLUT3
e
GLUT3 (SLC2A3)
Relative mRNA expression
M0
• sh-Con
• sh-GLUT3
• sh-GLUT3 - GLUT3 (WT)
• sh-GLUT3 + R331W
f
CXCL10
M0
M1
g
G3iA (μM)
IL-4
h
GLUT3 (SLC2A3)
i
CXCL10

Supplementary Figure 7. Genetic and chemical inhibition of GLUT3 transport function. (a) Western blot analysis of GLUT3 mutant alleles in Rat2 fibroblasts after lentiviral transduction of the indicated plasmid. HSP90, loading control. **(b)** Representative immunofluorescence stains of Flag-GLUT3 (green) and DAPI (blue) in vector, GLUT3 WT, GLUT3 sh1sh3 resistant, and GLUT3 R331W expressing Rat2 fibroblasts. **(c)** 2-Deoxy-D-glucose (2-DG) uptake in Rat2 fibroblasts expressing each plasmid. **(d)** Quantification of pSTAT6/tSTAT6 levels from WB of THP-1 cells treated with control or GLUT3 shRNA and the indicated GLUT3 allele (n=3 biological replicates). **(e-f)** Expression of SLC2A3 (e) or CXCL10 (f) was assessed in THP-1 after M2 (g) or M1 (h) induction (24 hours). **(g)** Quantification of pSTAT6/tSTAT6 levels from WB of THP-1 cells treated with indicated amount of GLUT3 inhibitor G3iA + IL-4 stimulation (n=3 biological replicates).**(h-i)** Expression of SLC2A3 (h) or CXCL10 (i) was assessed in THP-1 cells with the indicated concentration of G3iA +/- IL-4 stimulation (24 hours). P values were calculated by two-way ANOVA with Tukey's test (c-d, g-j) or one-way ANOVA with Dunnett's test (e-f) or *P < 0.05, **P < 0.01, ***P < 0.001.

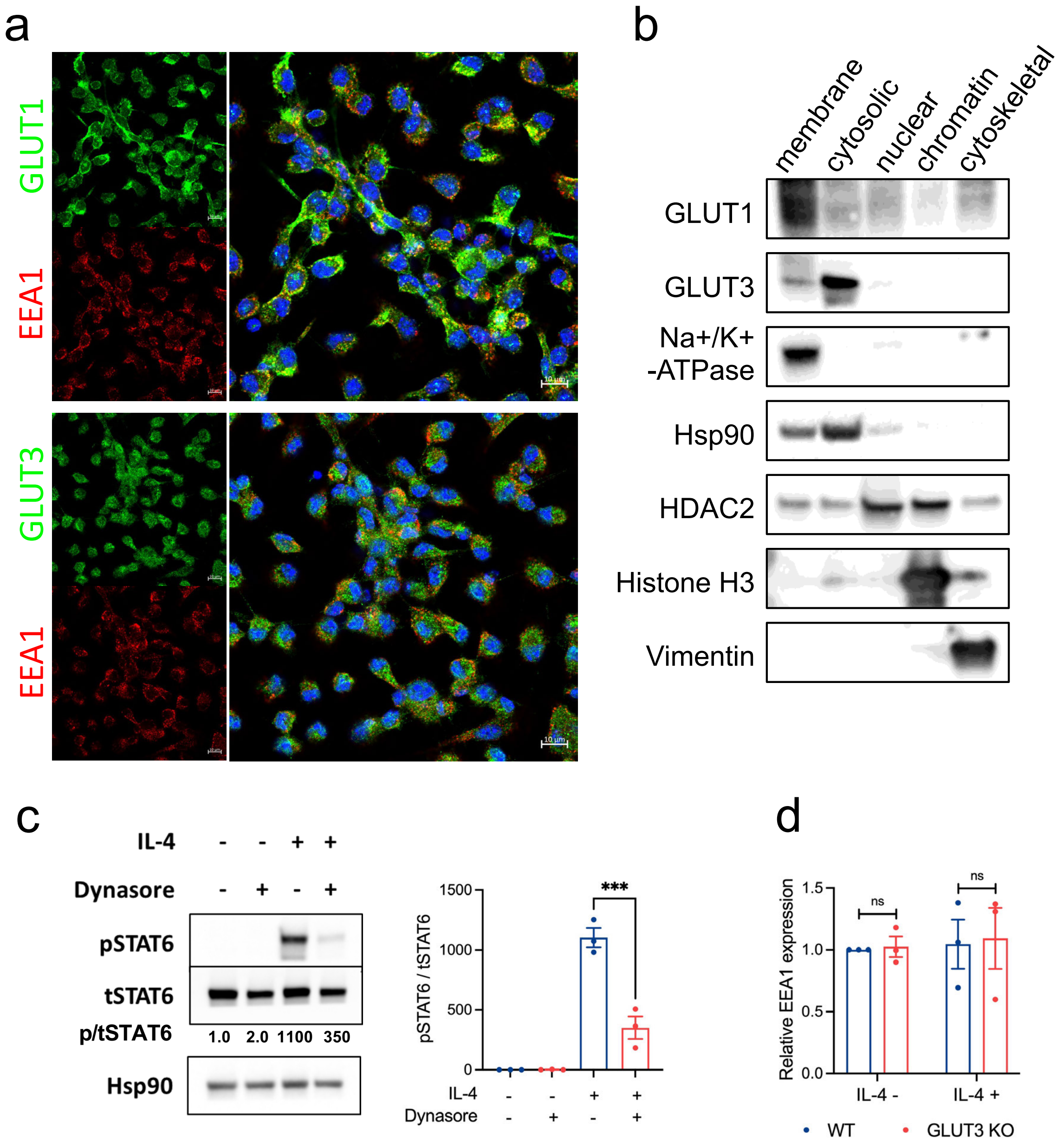

Supplementary Figure 8. Endosomal localization of GLUT3 promotes STAT6 activation. (a) Representative immunofluorescence image of BMDM labeled with GLUT1 (upper panel, green), GLUT3 (lower panel, green), F4/80 (red), and DAPI (blue). Scale bar =10 μm. **(b)** Western blot of GLUT1 and GLUT3 in membrane, cytosol, nuclear, chromatin, and cytoskeletal fraction from THP-1 cells. Na+K+-ATPase, Hsp90, HDAC2, Histone H3, and Vimentin, loading controls for fractionation, loading controls. **(c)** Quantification of pSTAT6/tSTAT6 levels from WB of THP-1 cells +/- Dynasore (endocytosis inhibitor) -/+ IL-4 stimulation (30 min) (n=3 biological replicates). **(c)** Western blot analysis of the expression of pSTAT6 and STAT6 in THP-1 cells +/- IL-4 (30 min) +/- Dynasore. Mean of pSTAT6/tSTAT6 levels from quantification of WB (n=3 biological replicates). **(d)** Quantification of EEA1 levels from WB of WT or GLUT3 KO BMDM -/+ IL-4 stimulation (30 min) (n=3 biological replicates). P values were calculated by two-way ANOVA with Tukey's test (c-d, g-j) or one-way ANOVA with Dunnett's test (e-f) or *P < 0.05, **P < 0.01, ***P < 0.001.

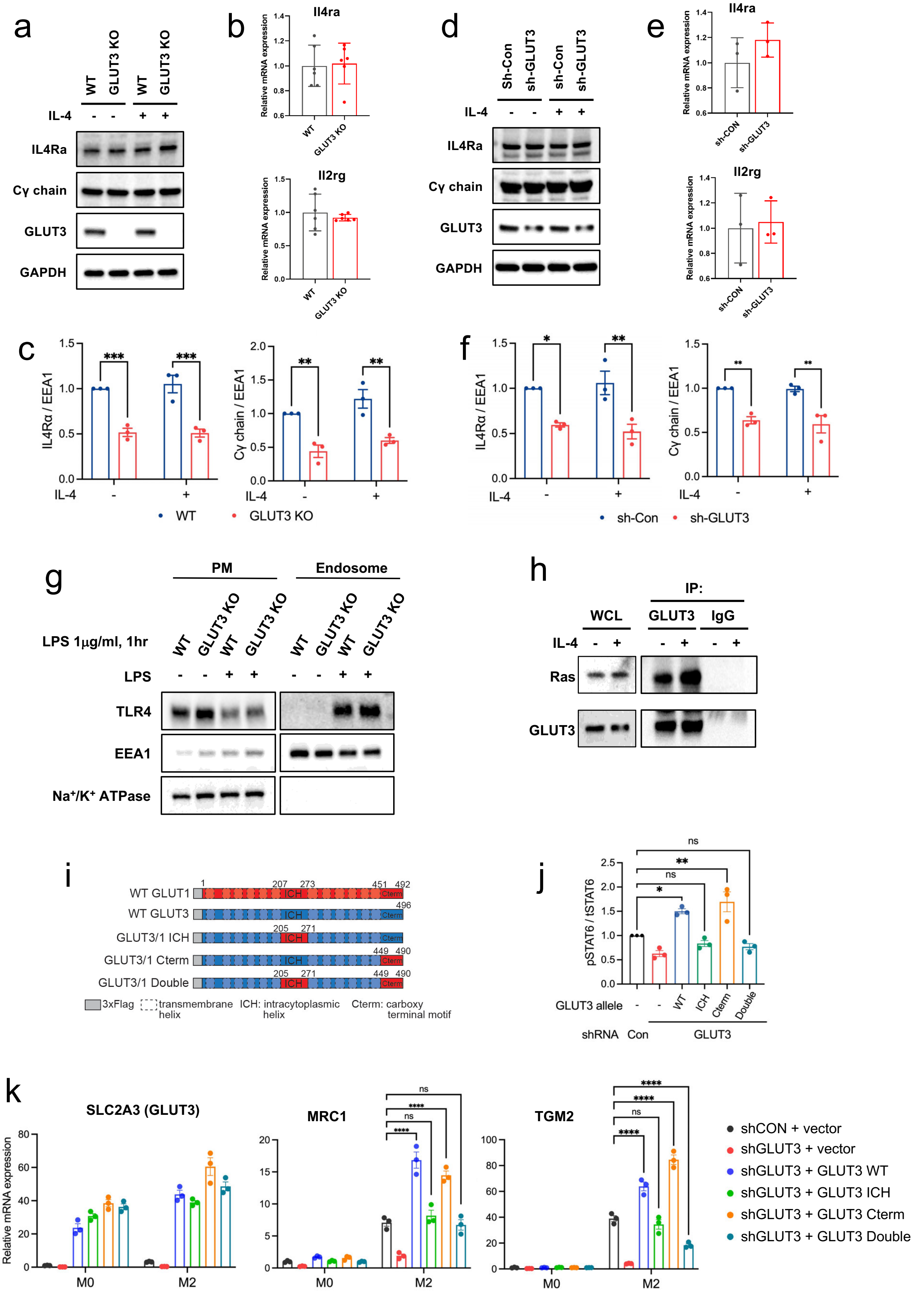
a
WT
GLUT3 KO
WT
GLUT3 KO
IL-4
IL4Ra
Cγ chain
GLUT3
GAPDH
b
Il4ra
Relative mRNA expression
WT
GLUT3 KO
Il2rg
d
Sh-Con
sh-GLUT3
sh-Con
sh-GLUT3
IL-4
IL4Ra
Cγ chain
GLUT3
GAPDH
e
Il4ra
Relative mRNA expression
sh-CON
sh-GLUT3
Il2rg
c
IL4Rα / EEA1
Cγ chain / EEA1
IL-4
WT
GLUT3 KO
f
IL4Rα / EEA1
Cγ chain / EEA1
IL-4
sh-Con
sh-GLUT3
g
PM
Endosome
LPS 1μg/ml, 1hr
WT
GLUT3 KO
LPS
TLR4
EEA1
Na+/K+ ATPase
h
IP:
WCL
GLUT3
IgG
IL-4
Ras
GLUT3
i
WT GLUT1
WT GLUT3
GLUT3/1 ICH
GLUT3/1 Cterm
GLUT3/1 Double
ICH
Cterm
3xFlag
transmembrane helix
ICH: intracytoplasmic helix
Cterm: carboxy terminal motif
j
pSTAT6 / tSTAT6
GLUT3 allele
WT
ICH
Cterm
Double
shRNA
Con
GLUT3
k
SLC2A3 (GLUT3)
MRC1
TGM2
Relative mRNA expression
M0
M2
shCON + vector
shGLUT3 + vector
shGLUT3 + GLUT3 WT
shGLUT3 + GLUT3 ICH
shGLUT3 + GLUT3 Cterm
shGLUT3 + GLUT3 Double

Supplementary Figure 9. GLUT3 promotes binds Ras and promotes IL4Ra endocytosis and M2 signaling through its ICH motif. (a) Western blot for IL4Rα and Cγ chain in WT and GLUT3 KO BMDMs. GAPDH, loading control. **(b)** mRNA levels of IL4Rα and Cγ chain transcripts in WT and GLUT3 KO BMDMs. **(c)** Quantification of IL4Rα or Cγ chain relative to EEA1 levels from WB of WT or GLUT3 KO BMDM (n=3 biological replicates). **(d)** Western blot for IL4Rα and Cγ chain in WT and GLUT3 KO IL4Rα and Cγ chain in THP-1 cells transduced with sh-Con or sh-GLUT3 plasmid. GAPDH, loading control. **(e)** mRNA levels of IL4Rα and Cγ chain transcripts in THP-1 cells transduced with shCon or shGLUT3. **(f)** Quantification of IL4Rα or Cγ chain relative to EEA1 levels from WB of THP-1 cells transduced with sh-Con or sh-GLUT3 -/+ IL-4 stimulation (30 min) (n=3 biological replicates). **(g)** Western blot of TLR4 in the plasma membrane (PM) and endosomal fractions from WT and GLUT3 KO BMDMs -/+ LPS stimulation. Na^+/K^+-ATPase and EEA1, fractionation controls. **(h)** Interaction between GLUT3 and Ras in WT BMDMs. GLUT3 was immunoprecipitated from the cell lysates and Ras (D2C1 Rabbit mAb recognizing N-Ras and K-Ras) was detected by Western blot. **(i)** Schematic of chimeric GLUT1/GLUT3 mutants used in Ras co-immunoprecipitation experiments. Alleles were amino-terminally tagged with a 3xFlag epitope tag. Dashed boxes indicated predicted transmembrane (TM) domains. **(j)** Quantification of pSTAT6/tSTAT6 levels from WB of THP-1 cells expressing indicated shRNA and GLUT3 allele or vector controls (n=3 biological replicates) + IL-4 stimulation (30 min) (n=3 biological replicates). **(k)** Expression of indicated gene was assessed in THP-1 cells expressing the indicated shRNA and GLUT3 allele or control +IL-4 stimulation (24 hours). P values were calculated by two-way ANOVA with Tukey's test (b, d-f, i) or one-way ANOVA with Dunnett's test (h) or $^*P < 0.05$, $^{**}P < 0.01$, $^{***}P < 0.001$.

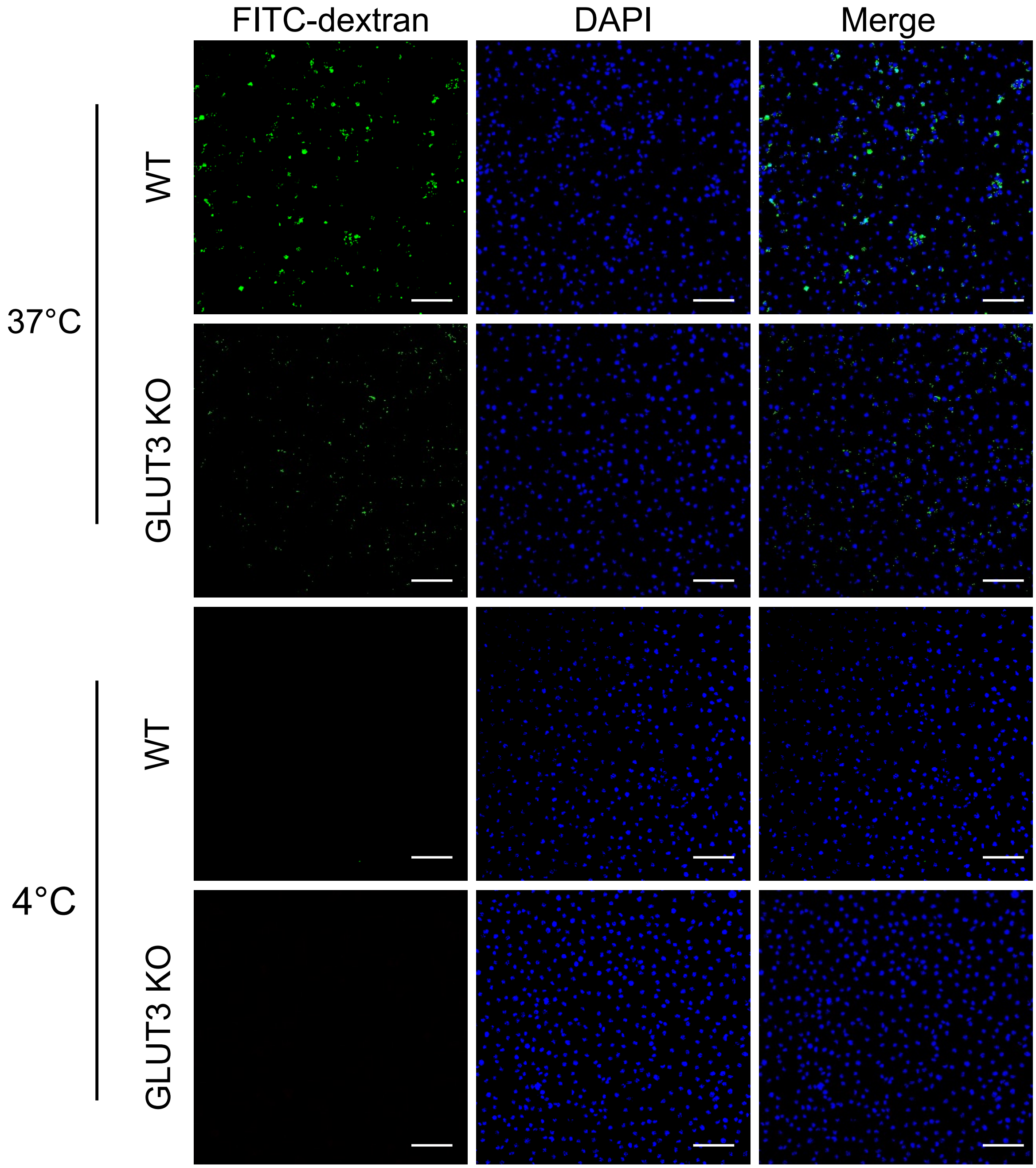

Supplementary Figure 10. Macropinocytosis. Representative IF images of WT or GLUT3 KO BMDMs incubated in FITC-Dextran at the indicated temperature. Absence of FITC-Dextran uptake at 4°C confirms fluorescence to be consistent with macropinocytosis. Scale bar=100μM. Quantification of mean fluorescence intensity per cell (n=3 biological replicates).

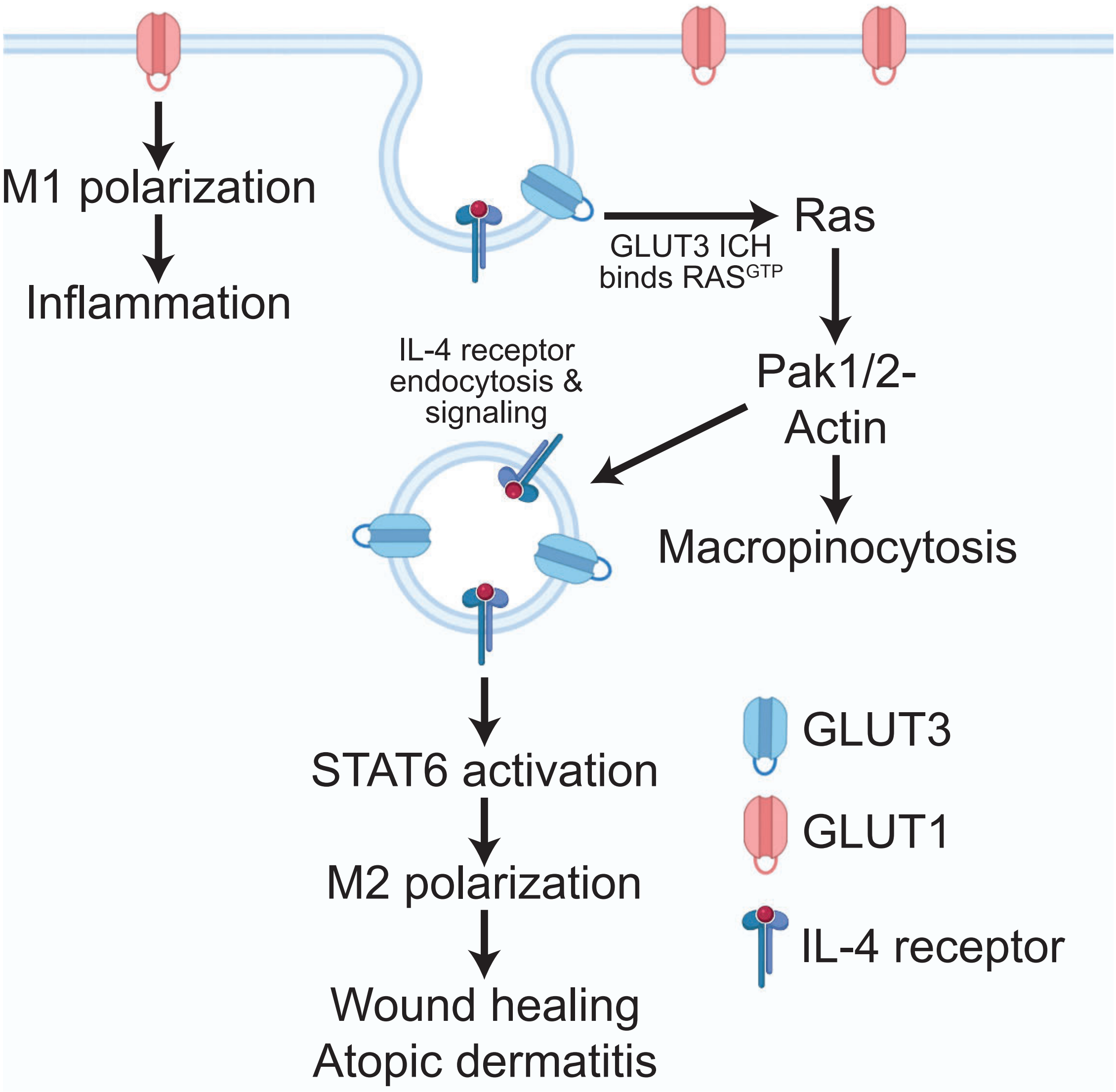

Supplementary Figure 11. Model of GLUT1 and GLUT3 function in macrophages.

Full unedited gel for Figure 1C

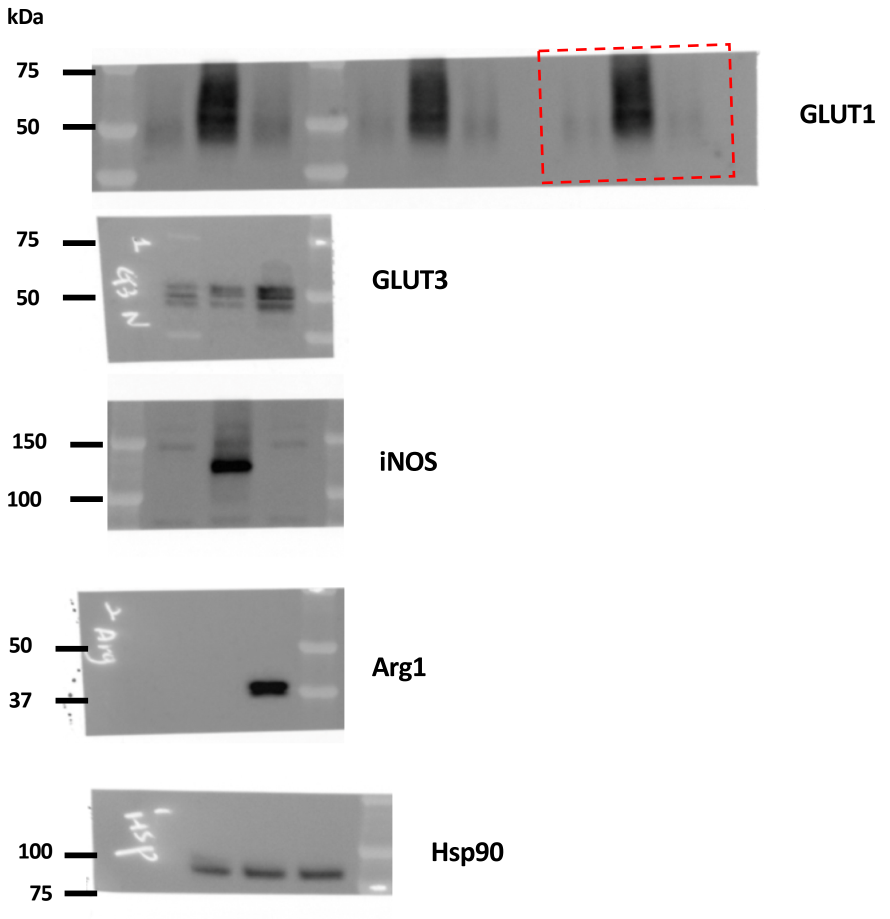

Full unedited gel for Figure 1D

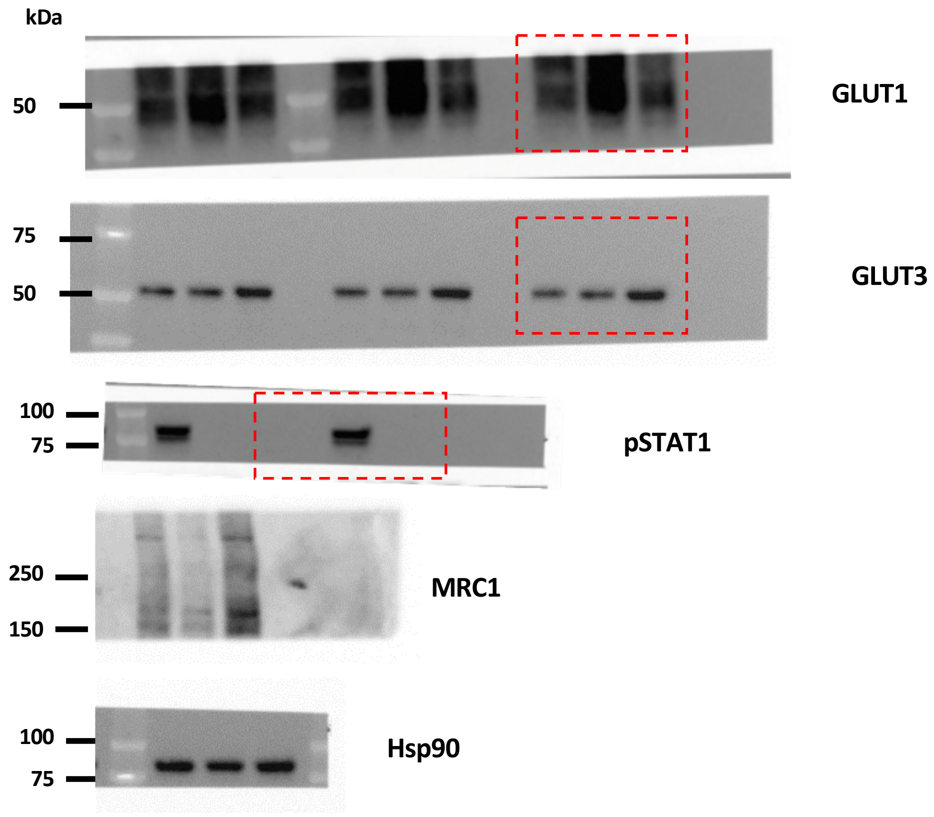

Full unedited gel for Figure 4A

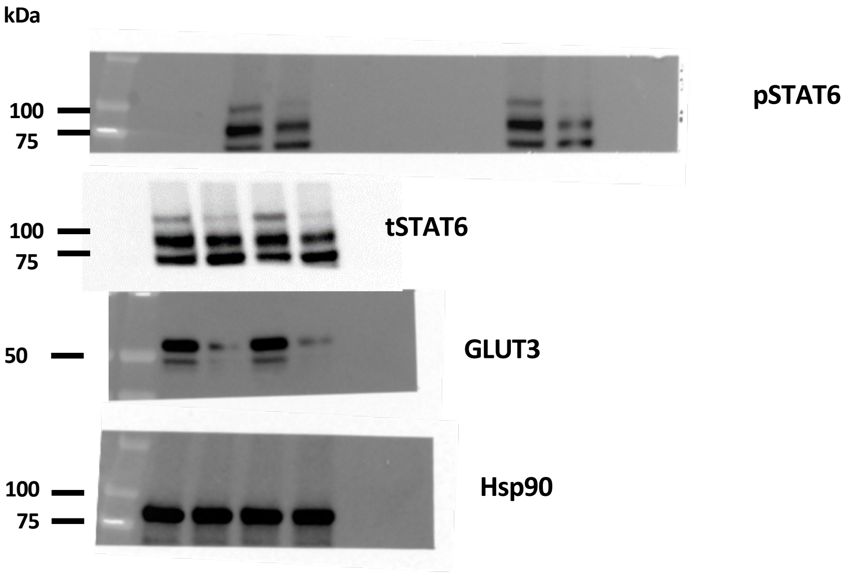

Full unedited gel for Figure 4B

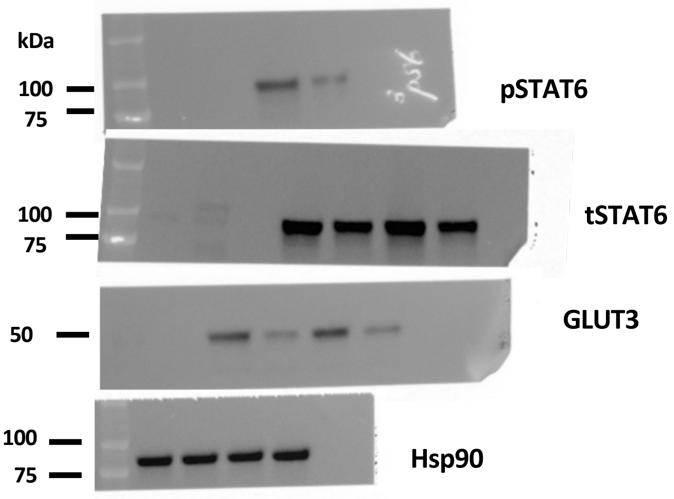

Full unedited gel for Figure 4C

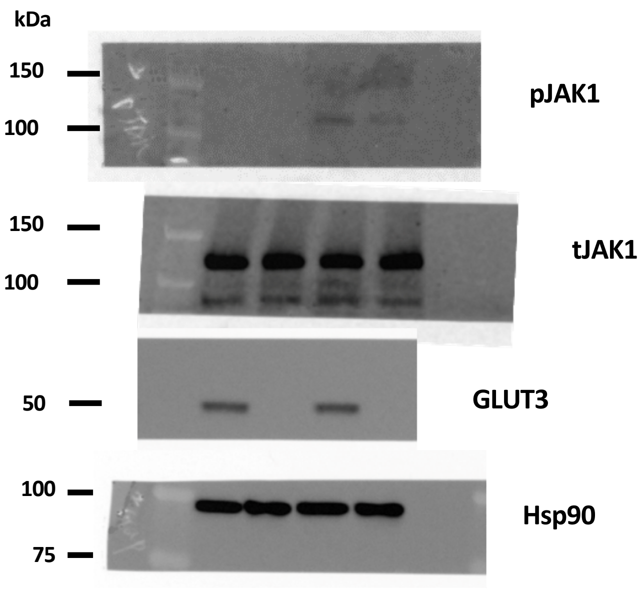

Full unedited gel for Figure 4D

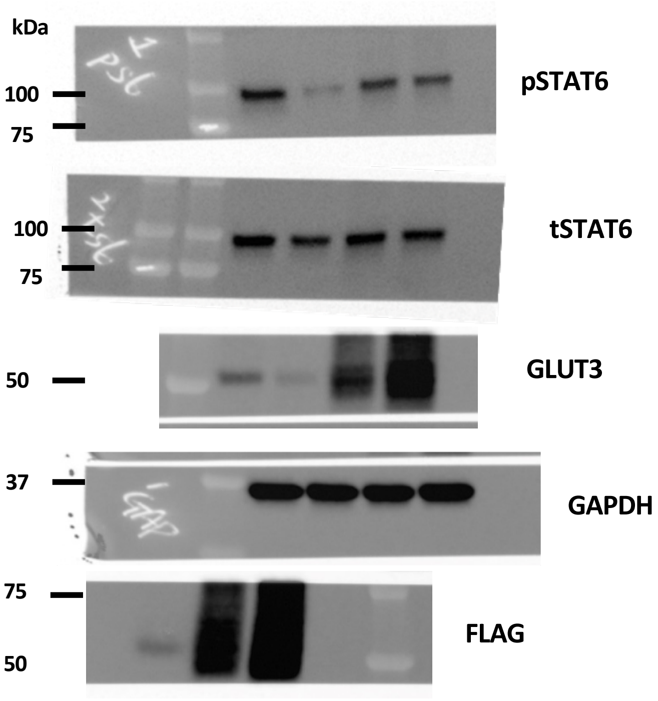

Full unedited gel for Figure 4F

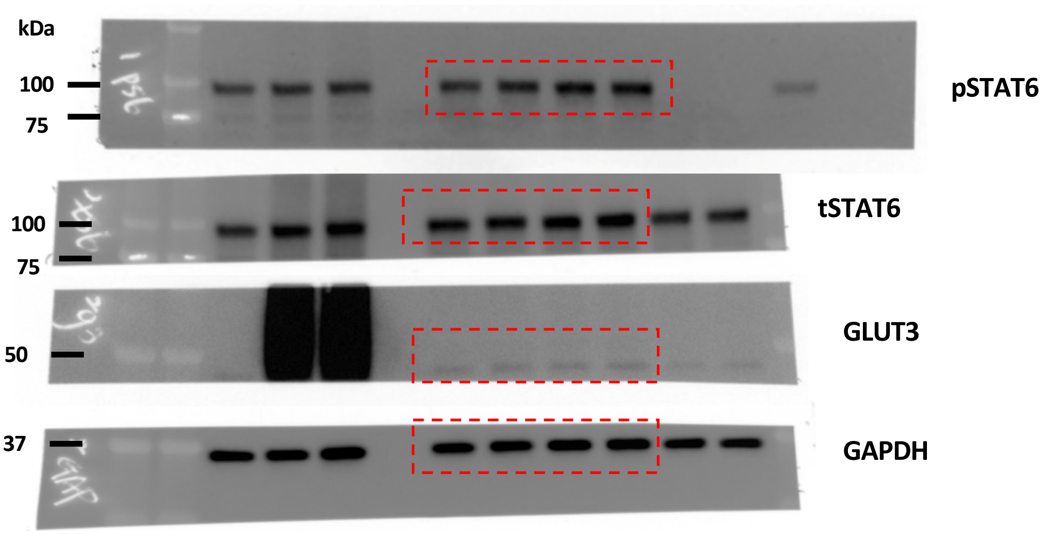

Full unedited gel for Figure 5B

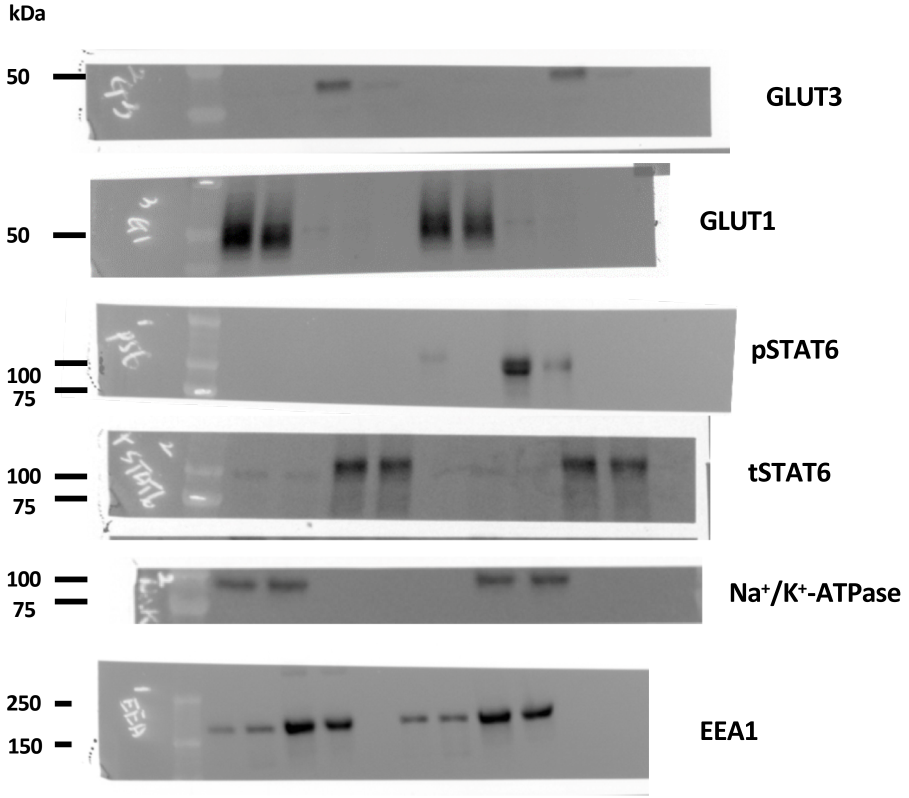

Full unedited gel for Figure 5C

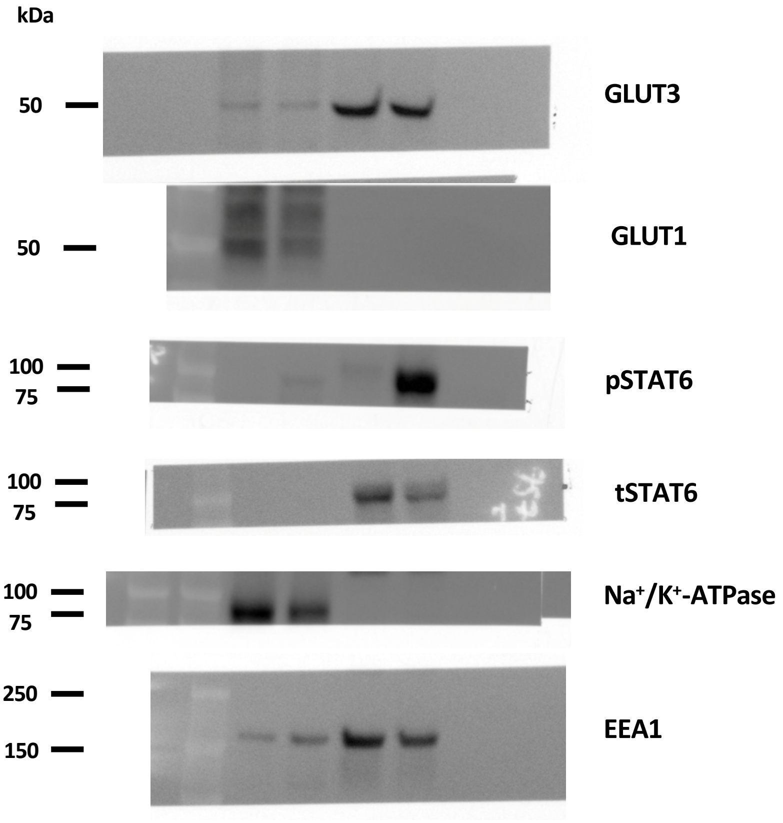

Full unedited gel for Figure 5D

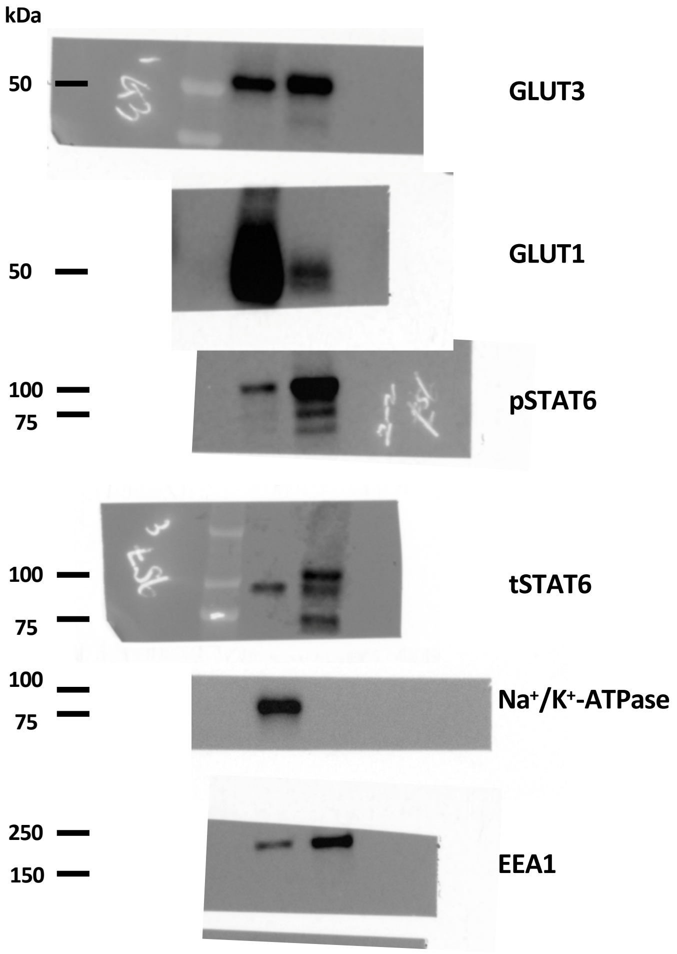

Full unedited gel for Figure 5E

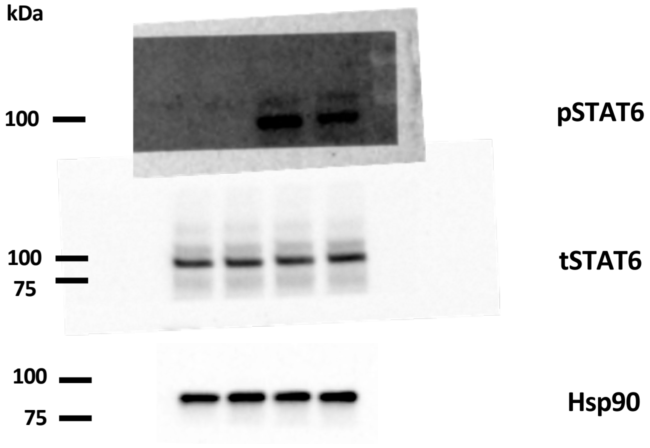

Full unedited gel for Figure 6A

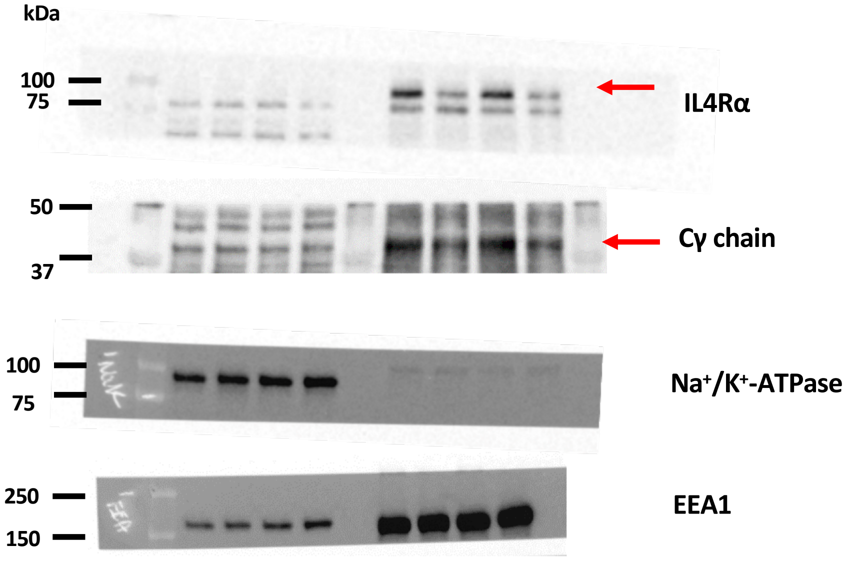

Full unedited gel for Figure 6B

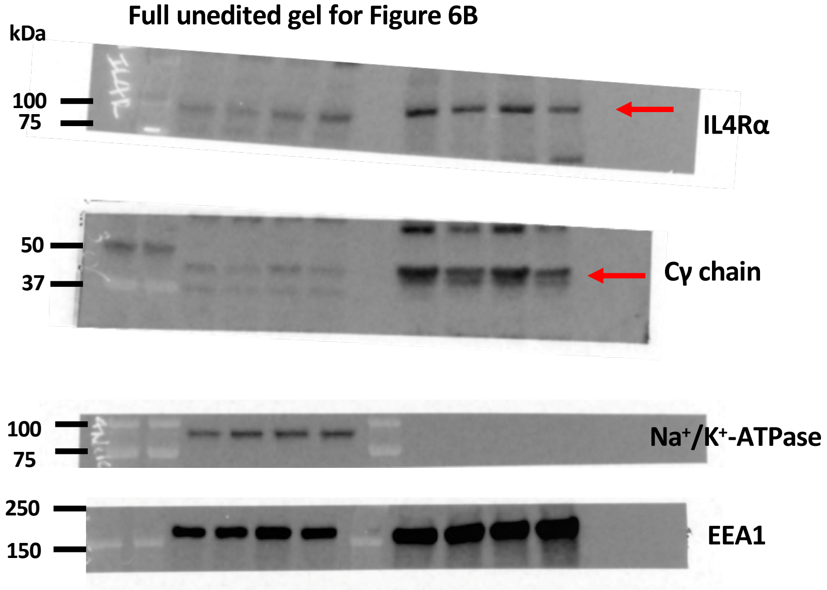

Full unedited gel for Figure 6C

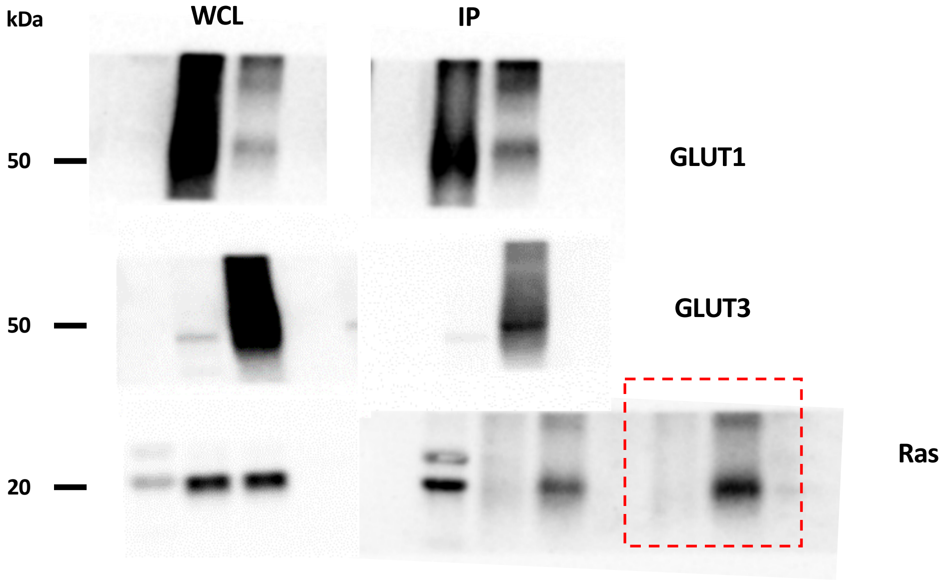

Full unedited gel for Figure 6D

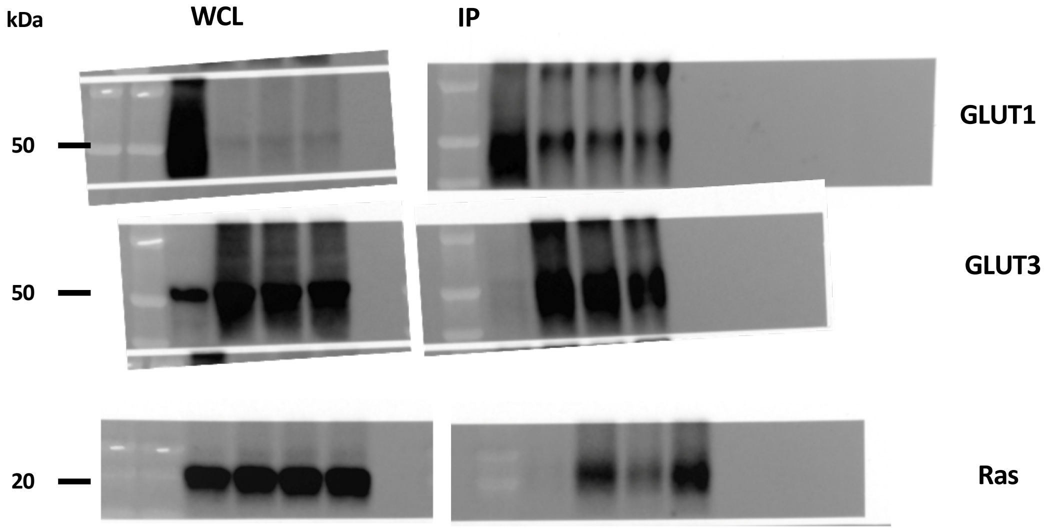

Full unedited gel for Figure 6E

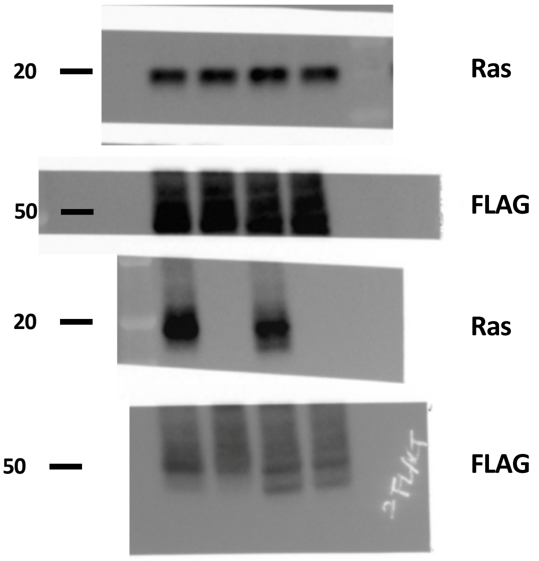

Full unedited gel for Figure 6F

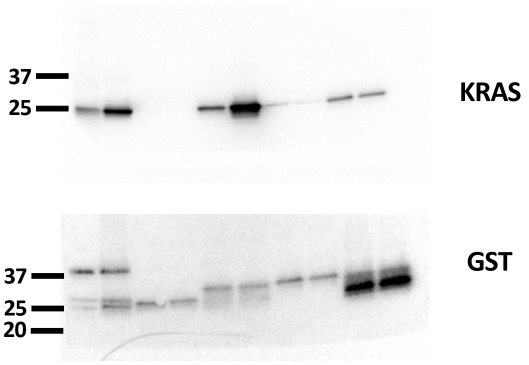

Full unedited gel for Figure 6G

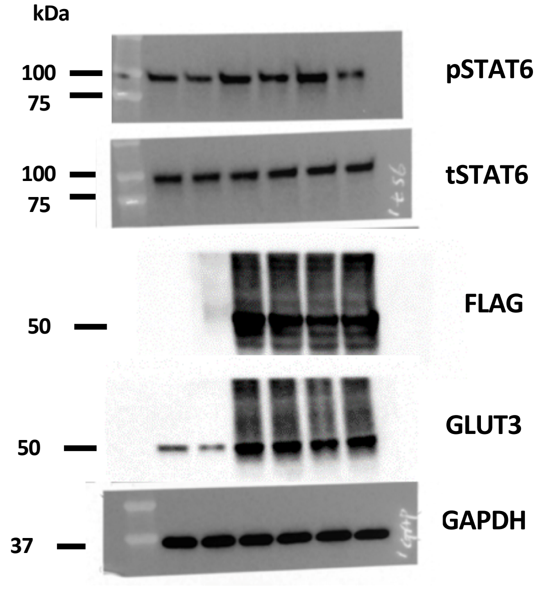

Full unedited gel for Figure 7A

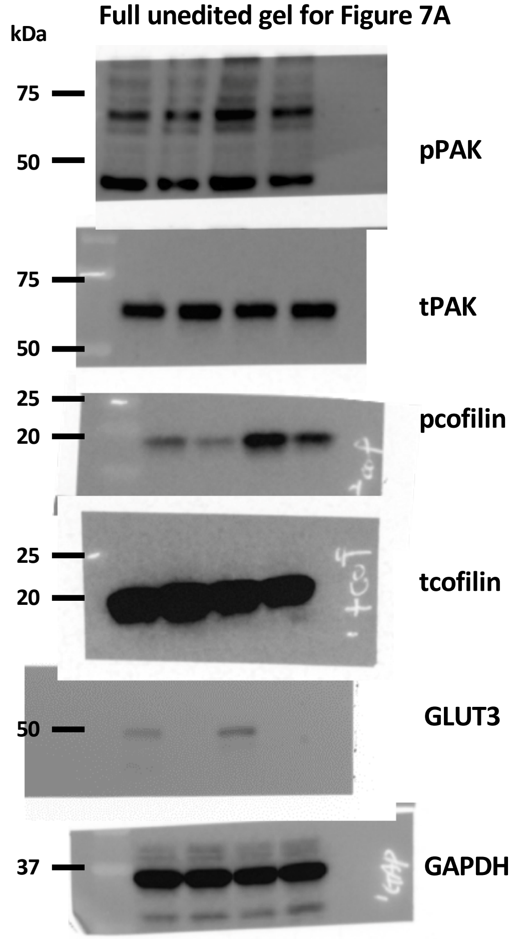

Full unedited gel for Figure 7B

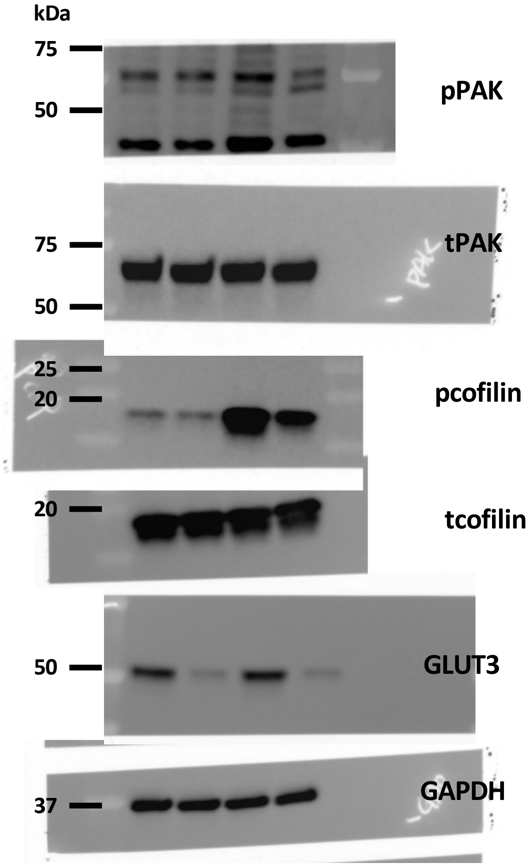

Full unedited gel for Figure 7C

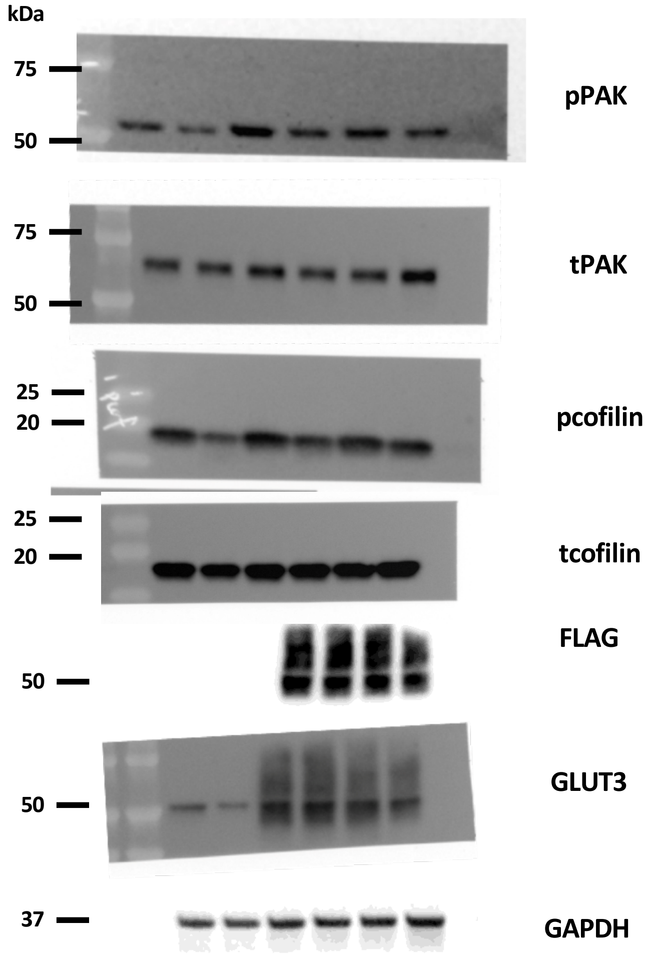

Full unedited gel for Supplementary Figure 6A

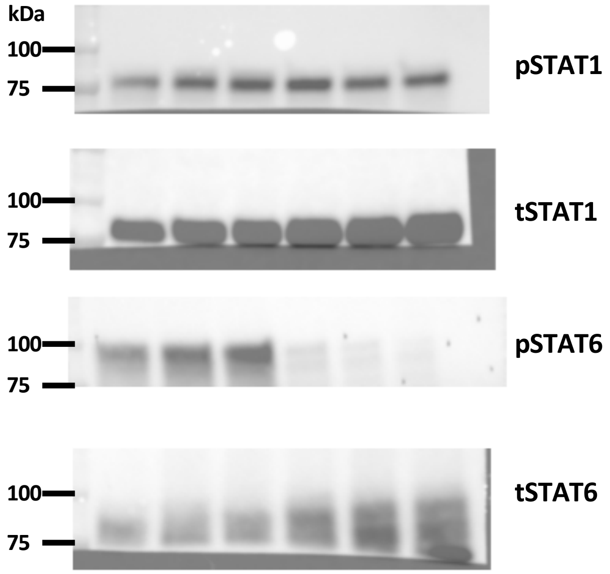

Full unedited gel for Supplementary Figure 6D

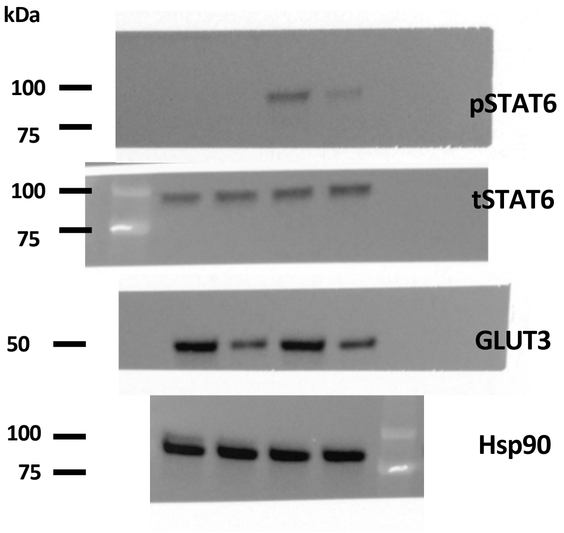

Full unedited gel for Supplementary Figure 6E

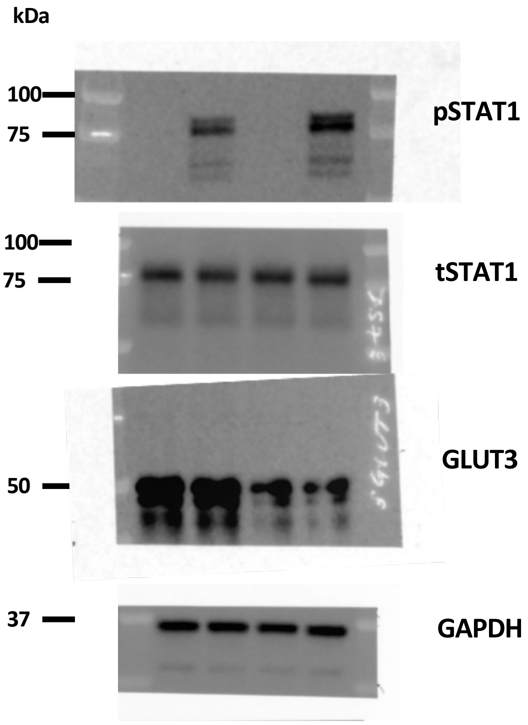

Full unedited gel for Supplementary Figure 7A

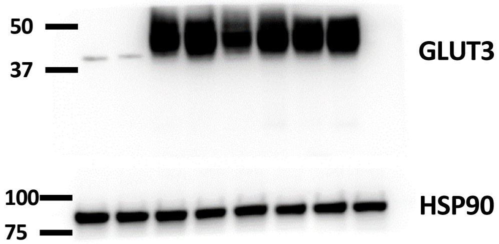

Full unedited gel for Supplementary Figure 8B

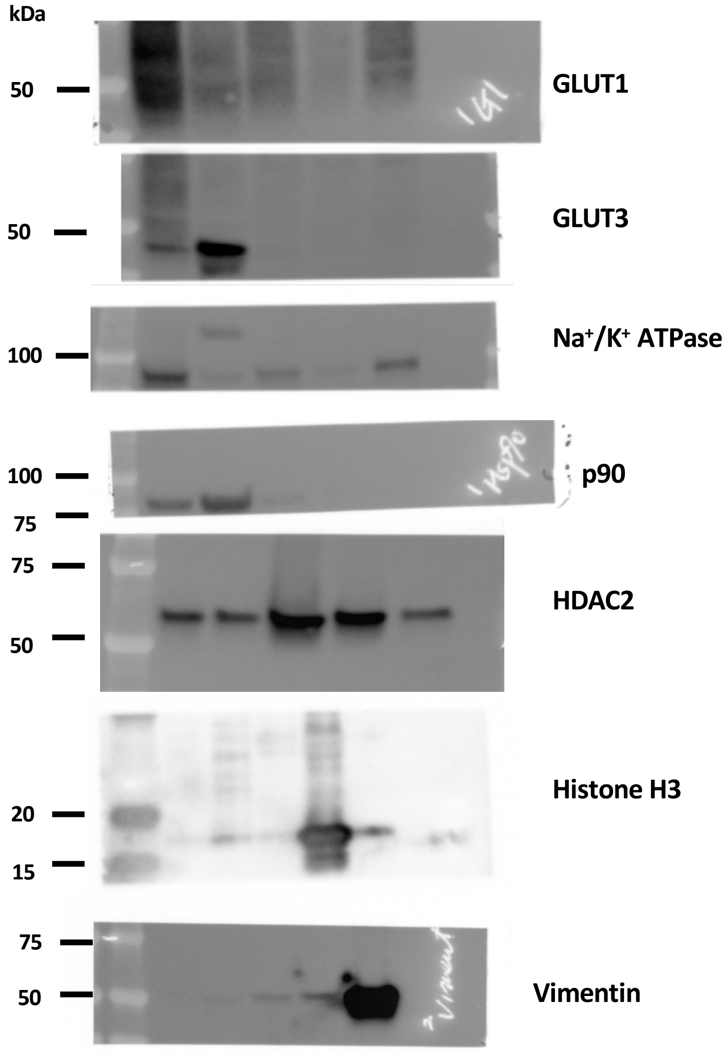

Full unedited gel for Supplementary Figure 8C

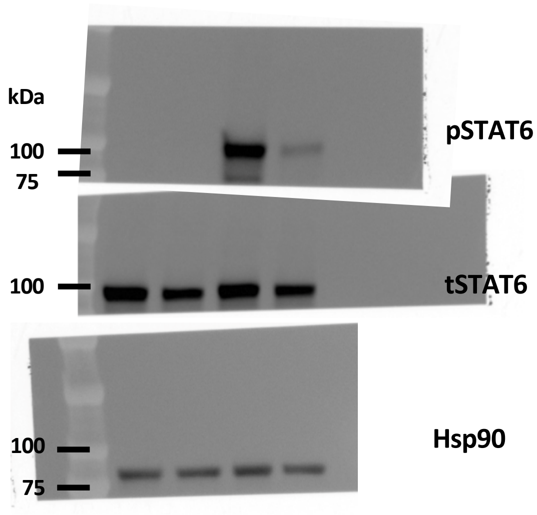

Full unedited gel for Supplementary Figure 9A

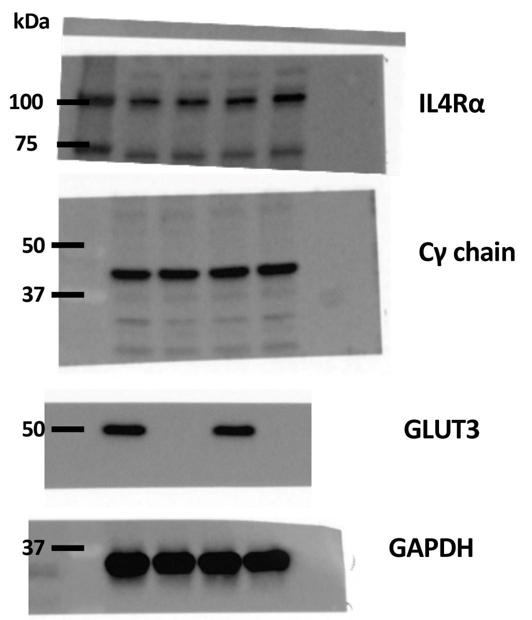

Full unedited gel for Supplementary Figure 9D

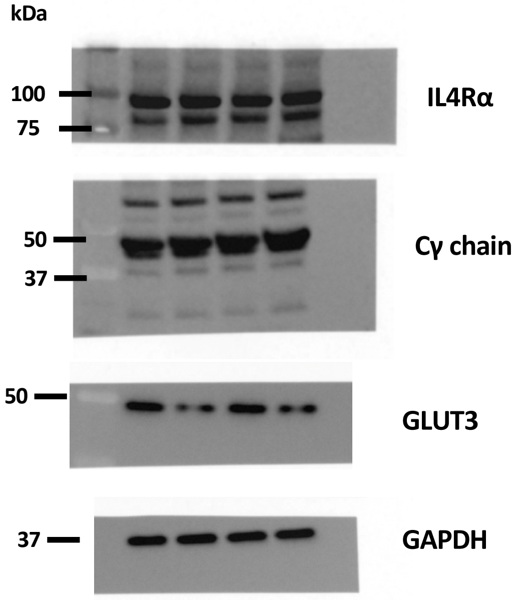

Full unedited gel for Supplementary Figure 9G

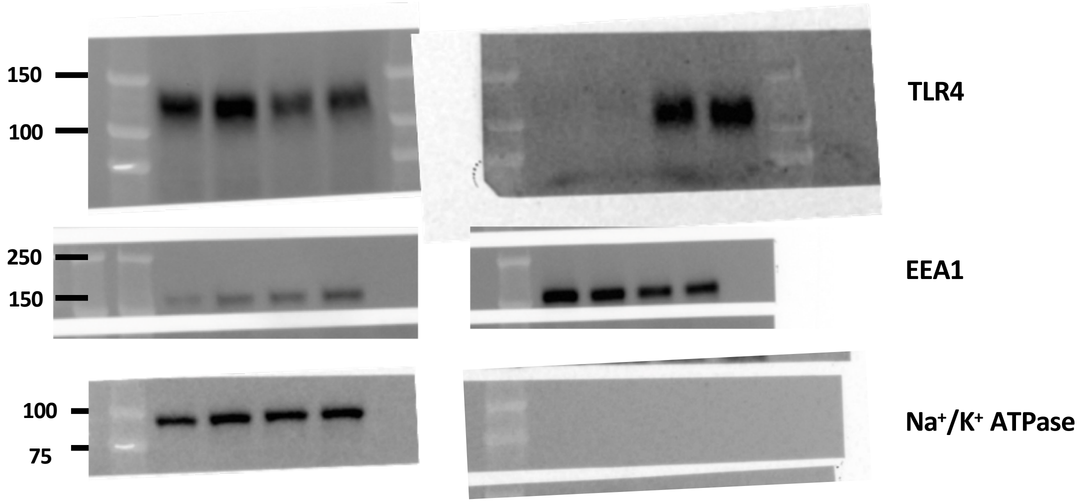

Full unedited gel for Supplementary Figure 9H

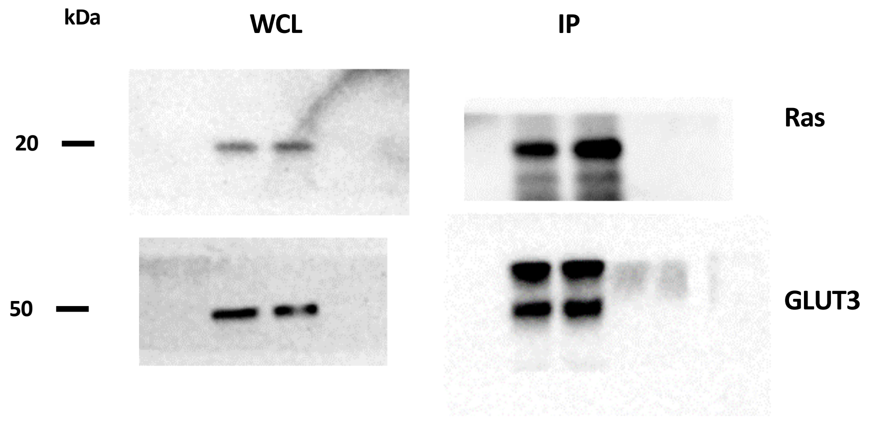

Supplement: Supplemental data [file jci-133-170706-s106.pdf]
